# Salt inducible kinases as novel Notch interactors in the developing *Drosophila* retina

**DOI:** 10.1371/journal.pone.0234744

**Published:** 2020-06-15

**Authors:** H. Bahar Şahin, Sercan Sayın, Maxine Holder, Kuyaş Buğra, Arzu Çelik

**Affiliations:** 1 Department of Molecular Biology and Genetics, Bogazici University, Bebek, Istanbul, Turkey; 2 Apoptosis and Proliferation Control Laboratory, The Francis Crick Institute, London, United Kingdom; 3 Center for Life Sciences and Technologies, Bogazici University, Bebek, Istanbul, Turkey; Simon Fraser University, CANADA

## Abstract

Developmental processes require strict regulation of proliferation, differentiation and patterning for the generation of final organ size. Aberrations in these fundamental events are critically important in tumorigenesis and cancer progression. *Salt inducible kinases* (*Siks*) are evolutionarily conserved genes involved in diverse biological processes, including salt sensing, metabolism, muscle, cartilage and bone formation, but their role in development remains largely unknown. Recent findings implicate Siks in mitotic control, and in both tumor suppression and progression. Using a tumor model in the *Drosophila* eye, we show that perturbation of Sik function exacerbates tumor-like tissue overgrowth and metastasis. Furthermore, we show that both *Drosophila Sik* genes, *Sik2* and *Sik3*, function in eye development processes. We propose that an important target of Siks may be the Notch signaling pathway, as we demonstrate genetic interaction between Siks and Notch pathway members. Finally, we investigate Sik expression in the developing retina and show that Sik2 is expressed in all photoreceptors, basal to cell junctions, while Sik3 appears to be expressed specifically in R3/R4 cells in the developing eye. Combined, our data suggest that *Sik* genes are important for eye tissue specification and growth, and that their dysregulation may contribute to tumor formation.

## Introduction

The generation of tissues and the establishment of cellular diversity in multicellular organisms relies upon cell-cell communication during development. One of the prominent and evolutionarily-conserved signaling pathways that regulates specification and patterning of tissues is the Notch signaling pathway. Notch has been established as a key regulator of development in both vertebrates and invertebrates (reviewed in [1]). Interestingly, many signaling pathways are involved in cell fate determination, including the Notch pathway, have been implicated in promoting or suppressing cancer when mutated or misregulated [2].

The *Drosophila* eye has been used as a model to study development, patterning, and tumor growth. The eye starts to develop in the second instar larval stage, from the eye-antennal imaginal disc, a single-layer epithelium. Notch signaling is critical during the early stages of eye development. The posterior side of the disc is specified as eye primordium, marked by Notch expression, while the anterior is specified as antenna and is marked by EGFR expression [3] (S1A Fig). During the late second instar stage, Notch expressed by the organizer midline cells is critical for establishing the dorsal-ventral (D-V) polarity of the eye disc. The Notch receptor is induced by its ligands Delta on the dorsal side, and Serrate on the ventral side [4]. The role of the glycosyltransferase Fringe is to modify Notch and inhibit its activation in the ventral half except the midline [5] (S1B Fig). In the late third instar stage, a differentiation wave known as the morphogenetic furrow (MF) moves from posterior to the anterior of the disc (S1C Fig), leaving differentiated cells behind. Photoreceptors (PRs) and accessory cells are specified sequentially from undifferentiated cells, with each step requiring Notch signaling [6]. Once the neural and nonneural cells complete differentiation and planar cell polarity is established, the PRs start to elongate in the apical-basal axis and change their morphology, while remaining firmly attached to each other via adherens junctions (AJs), containing Cadherin protein (reviewed in [7]). This coordinated differentiation and organization of cells gives rise to an adult eye that is composed of ~800 ommatidia, each containing eight PRs, plus supporting cone, pigment, and bristle cells (reviewed in [8]).

Salt-inducible kinases (Siks) represent an evolutionarily conserved Ser/Thr kinase family of proteins that belong to the AMP-activated protein kinase (AMPK) superfamily. The two fly homologs (Sik2 and Sik3) show the same protein organization and high similarity with their human counterparts (SIK1, SIK2, SIK3). The kinase domains are well conserved (>85% similarity between human and fly, at the protein level) and a point mutation in this domain, homologous to the K170 residue of fly Sik2, leads to inactivation of the kinase domain (kinase-dead allele) [9] (S2A and S5 Figs).

Sik enzymatic activity is regulated through a number of phosphorylation sites, by two main signals; Lkb-1, a general tumor suppressor, and PKA, an effector of second messenger cAMP. Siks are not catalytically active until phosphorylated by Lkb-1 on their T-loop [10]. Phosphorylation by PKA is inhibitory; and mutation of the target serine to an alanine primes the Sik protein for over-activity and results in constitutively active Sik alleles, such as S1032A in fly Sik2 or S563A in fly Sik3 (S2A and S5 Figs) [11]. The well-characterized downstream effectors of Siks are CREB (cAMP Response Element Binding Protein), CREB-regulated transcription coactivator proteins (CRTCs / TORCs), HDACs, and IRS-1 [12].

Siks are involved in diverse biological processes including sugar and lipid metabolism [13–18], starvation response [9, 19–21], neuronal and glial survival [22, 23], autophagy [24], mitotic control [25] and regulation of sleep and the circadian clock [26, 27]. Although these kinases have been extensively studied in metabolism and homeostasis, their function during development are less clear. So far, Siks have been shown to regulate body size in *C*. *elegans* [28] and were linked to regulation of tissue growth and cell polarity regulators in *Drosophila* [29]. Additionally, both *Drosophila* Sik2 and Sik3 were shown to interact with the Hippo pathway to regulate tissue growth and size [30]. Siks are also implicated in diseases such as diabetes [31], epilepsy [32], various cancer types [33–36] and cancer progression (reviewed in [37]). Given their proposed roles in mitotic mechanisms and response to starvation, Siks have been shown to be the molecular link between sugar metabolism, tumorigenesis and cancer progression [38, 39].

Here, we make use of a *Drosophila* tumor model in the retina to investigate whether Siks can induce tumor progression. Our results indicate that Siks are expressed in the developing eye disc, and that Sik function is required to establish proper eye size and retinal development. Furthermore, we show a genetic interaction between Siks and the Notch signaling pathway, and that increased or decreased activity of Sik promotes Notch-associated tumor-like growth of eye tissue. This suggests that Notch pathway members could represent direct or indirect downstream targets of Siks.

## Materials and methods

### Fly genetics and husbandry

Flies were raised at 25°C, on regular fly food. *Sik2*^*Δ41*^ and *UAS-Sik2*^*K170M*^ lines were kindly provided by Jongkyeong Chung (Seoul, Korea) [9]. Sensitized and eyeful lines were kindly provided by Bassem Hassan (Paris, France) [2]. *UAS-Dicer2; ey-GAL4*, *lGMR-Gal4 / CyO* was a kind gift from Claude Desplan (New York, USA) [40].

The following fly lines were obtained from the Bloomington *Drosophila* Stock Center: *w*^*1118*^ (3605), *UAS-Notch* (26820), *UAS-Delta* (5612), *UAS-Ser* (5815), *UAS-Fringe* (5831), *UAS-Delta*.*DN* (26697), *lGMR-GAL4* (8121), *ey-GAL4* (5535), *elav-GAL4* (458), *mirr-GAL4* (29650), *sca-GAL4* (6479), *neoFRT42D*, *GMR-myr-GFP* (7110), *neoFRT42D*, *GMR-hid / CyO; ey-GAL4*, *UAS-FLP* (5251), *actin-GAL4* (25374). Following RNAi lines were supplied by the VDRC Stock Center: *Sik3-RNAi* (107458), *Sik2-RNAi* (103739), *Ser-RNAi* (108348), *Delta-RNAi* (3720), *Fringe-RNAi* (51977).

### Generation of fly lines

The *UAS-Sik3*::*T2A*::*mCherry* construct was generated with the full-length cDNA of *Sik3* isoform A. The pUAST-attB vector was modified to include the T2A::mCherry fusion protein. The T2A::mCherry plasmid was a kind gift from Dr. Stefan Fuss (Istanbul, Turkey). It was cloned into the pUAST-attB cloning site using the restriction enzymes *Xho*I and *Kpn*I. The cDNA template FBcl0162999, corresponding to *Sik3*, was purchased from *Drosophila* Genomics Resource Center (DGRC). *Sik3* cDNA was amplified by high fidelity DNA polymerase Advantage 2 (Clontech) and cloned into pUAST-attB-T2A::mCherry using the *Not*I and *Xho*I restriction sites, T2A and mCherry being N-terminal. Transgenic fly line was generated at Genetivision, Inc. (Houston, USA).

*Sik3*^*Δ109*^ allele was generated by excision of P element *P{EPgy2}CG42856*^*[EY14354]*^. Mobilization created a deletion of 9725 bp from exon 2 to exon 10. *CG15071* gene, which lies in the intron 2 of *Sik3* was completely deleted, while *CG42855* gene, which overlaps with the 5' of *Sik3* gene remained intact (S2A Fig).

### Recombinase mediated recombineering

BAC clones 149A06 for *Sik2* and 12M10 for *Sik3* genes were ordered from Berkeley *Drosophila* Genome Project (BDGP) in attB-P[acman]-Cm^R^-BW BAC vectors. The clones include the entire coding sequence, untranslated regions, 14 kb and 6 kb upstream regulatory sequence for *Sik2* and *Sik3* genes, respectively. Isoform A (CG42856-RA / NM_137515) was chosen for tagging the *Sik3* gene; *Sik2* has no alternative transcripts. The BAC clones were transformed into the SW102 strain of *E*. *coli*. In the first round of homologous recombination, the *galactokinase* gene (*GalK*) was inserted immediately upstream of the stop codon, as described previously [41]. Next, the *GalK* gene was replaced with *GFP* to tag *Sik2* and *mCherry* to tag *Sik3*. *GFP* and *mCherry* coding sequences were amplified by PCR, via primers containing homology arms (primers to recombine *GFP* to *Sik2*: CGCTCAGCGAGAGCCCCATCCTGGAGATATCGGAGCACCTTGAGTCAGTCGGCAGCGGCATGGTGAGCAAGGGCGAGGAG, and CTCCCATTCGAATTGCCCCAACCCCTCCACCGAATAACATCACACACGGCCTTACTTGTACAGCTCGTCCATG, primers to recombine *mCherry* to *Sik3*: GATATGCCAGCAGTTGATAAGCACCATCACCATGCAGCAGGTGGCAGGCGGCAGCGGCATGGTGAGCAAGGGCGAGGAG, and CAAGGCGAGTAGTCATTTTGGGTAGCTAGCAGGGCGGGATTCCATAATTTACTTGTACAGCTCGTCCATGC). A 3 amino acid linker (Gly-Ser-Gly) was inserted between *Sik* and the fluorescent genes to allow independent folding of the proteins [41]. Transgenic flies were generated by GenetiVision, Inc. (Houston, USA); tagged Sik2 and Sik3 constructs were targeted to genomic locations VK31(3L)62E1 and VK37(2L)22A3, respectively.

### Generation of tissue specific clones

Mitotic eye clones, marked by the absence of GFP, were generated by crossing *ey-FLP*.*N;; FRT42D P[GMR-myrGFP]* flies to *FRT42D*, *Sik3*^*Δ109*^. To generate *Sik3*^*Δ109*^ eye clones that are composed of mitotic clones only, *ey-GAL4*, *UAS-FLP1;; FRT42D*, *GMR-hid* flies were crossed to *FRT42D*, *Sik3*^*Δ109*^ flies. *Sik3*^*Δ109*^ mutant tissue was marked by the absence of *w*^+^.

### Immunohistochemistry

Whole mount of 0.5 μm-thick sectioned eye imaginal discs were dissected in cold PBS and fixed in 4% formaldehyde, at room temperature for 20 minutes. After 3 washes in PBX (0.3% TritonX-100 in 1X PBS) the discs were blocked for 1 hour at room temperature in BNT Buffer (10% donkey serum, 0.01% BSA, 250 mM NaCl, 0.1% Tween-20, PBS 1X). Eye imaginal discs were incubated with primary antibodies overnight at 4°C. After 3 washes in PBX, the discs were incubated with secondary antibodies for 2 hours at room temperature, and after several washing steps, they were mounted in Vectashield^©^ mounting medium.

Antibodies were used at the following concentrations: rabbit α-SIK3 human, polyclonal (Abcam ab88495, 1:50 for IHC, 1:500 for WB), mouse α-actin human, monoclonal (Cell Signaling 8H10D10, 1:1000 for WB), rabbit α-DsRed, polyclonal (Clontech 632496, 1:500 for WB, 1:100 for IHC, to detect mCherry), chicken α-GFP, polyclonal (Abcam ab13970, 1:1500 for IHC), Guinea pig α-Senseless *Drosophila* (kindly provided by H. Bellen, Houston, USA, 1:500 for IHC), rabbit α-Spalt (Salm) (kindly provided by F. Schnorrer, Marseille, France, 1:150 for IHC), mouse α-Seven-up (kindly provided by Y. Hiromi, Shizuoka, Japan, 1:500 for IHC), rat α-DNCad *Drosophila*, monoclonal (DSHB DN-Ex#8, 1:20 for IHC), mouse α-Armadillo *Drosophila*, monoclonal (DSHB N2-7A1, 1:50 for IHC), mouse α-rough *Drosophila*, monoclonal (DSHB 62C2A8, 1:50 for IHC), mouse α-Prospero *Drosophila*, monoclonal (DSHB MR1A, 1:5 for IHC), mouse α-Delta *Drosophila*, monoclonal (DSHB C594.9B, 1:50 for IHC), rat α-elav *Drosophila*, monoclonal (DSHB 7E8A10, 1:20 for IHC). All secondary antibodies except for donkey α-chicken FITC (Thermo Fischer, 1:800) were Alexa-conjugated (donkey α-rabbit Alexa 555, donkey α-mouse Alexa 488, donkey α-rat Alexa 647, goat α-guinea pig Alexa 555, goat α-mouse Alexa 555) (Molecular Probes, 1:800).

### Imaging / Electron microscopy

Immunohistochemistry (IHC) sample images were acquired using a Confocal TCS SP5 system. The whole mount fly images were acquired with a Leica DFC310 FX system, after samples were mounted in pure ethanol. Detailed whole mount fly images were acquired with a SEM (Scanning Electron Microscopy), by Boğaziçi University Advanced Technologies Research and Development Center, Electron Microscopy Facility. Gold coating was not applied.

### Protein analyses

Crude protein was extracted from dissected fly tissues in following buffer: 50 mM NaCl, 50 mM Tris-Cl pH 7.5, 10% Glycerol, 320 mM Sucrose, 1% Triton-X100, Complete Protease Inhibitor Cocktail^©^ Inhibitor (Roche). The tissues were homogenized and centrifuged for 10 minutes at 13,200 rpm, 4°C. The crude protein was loaded on an 8% SDS-PAGE, after denaturation by DTT. Separated proteins were transferred to a PVDF membrane with a pore size of 0.45 μm. The membrane was blocked in 5% non-fat milk in TBS-T (20 mM Tris-Cl pH 7.6, 150 mM NaCl, 0.1% Tween-20) and blotted with primary antibody in 5% milk solution, overnight at 4°C. Signals were detected by LumiGLO^®^ Reagent (Cell Signaling).

### Bioinformatic analyses

The protein sequences were downloaded from UniProtKB (http://www.uniprot.org/) and Protein similarity was calculated by EBI global protein alignment tool, Needle (EMBOSS) (http://www.ebi.ac.uk/Tools/psa/emboss_needle/), by pairwise comparison of the protein sequences of interest.

To establish the recognition pattern for salt inducible kinases, known Sik target proteins were chosen manually from publications. Only the experimentally proven target sequences were selected. Targets of different Sik homologs were pooled together (UniprotKB numbers are given in parentheses): *Drosophila* HDAC4 (Q9VYF3) [21], mouse HDAC4 (Q6NZM9) [42], mouse HDAC5, (Q9Z2V6) [43], *C*. *elegans* HDA-4 (O17323) [44], *Drosophila* CRTC (M9FPU2) [9], human CRTC1 (Q6UUV9) [45], mouse CRTC2 (Q3U182) [11], human CRTC3 (Q6UUV7) [46], *Drosophila* Sav (Q9VCR6) [30], human p35 (Q15078) [47], human p85α (P27986) [48], mouse CBP/p300 (Q09472) [49], human IRS-1 (P35568) [12], human tau (P10636) [50], human SMRT (Q9Y618) [51] and Sakamototide^®^ (synthetic peptide) [52]. Since *C*. *elegans* and *D*. *melanogaster* protein targets are missing it, the S/T at position -2 of the phosphorylation motif was ignored. The target sequences were aligned via EBI MView multiple alignment viewer using the default parameters (http://www.ebi.ac.uk/Tools/msa/mview/) [53]. Consensus sequence of 80% was accepted as the motif. The common pattern was searched against the whole *Drosophila melanogaster* proteome, by making use of the ScanProsite tool of ExPaSy (http://prosite.expasy.org/scanprosite/), using default parameters. This tool returned 507 hits; 307 were reviewed UniProtKB entries. Known targets and novel candidates were analyzed in the results.

## Results

### Sik proteins regulate growth in a *Drosophila* tumor model

**Fig 1:**
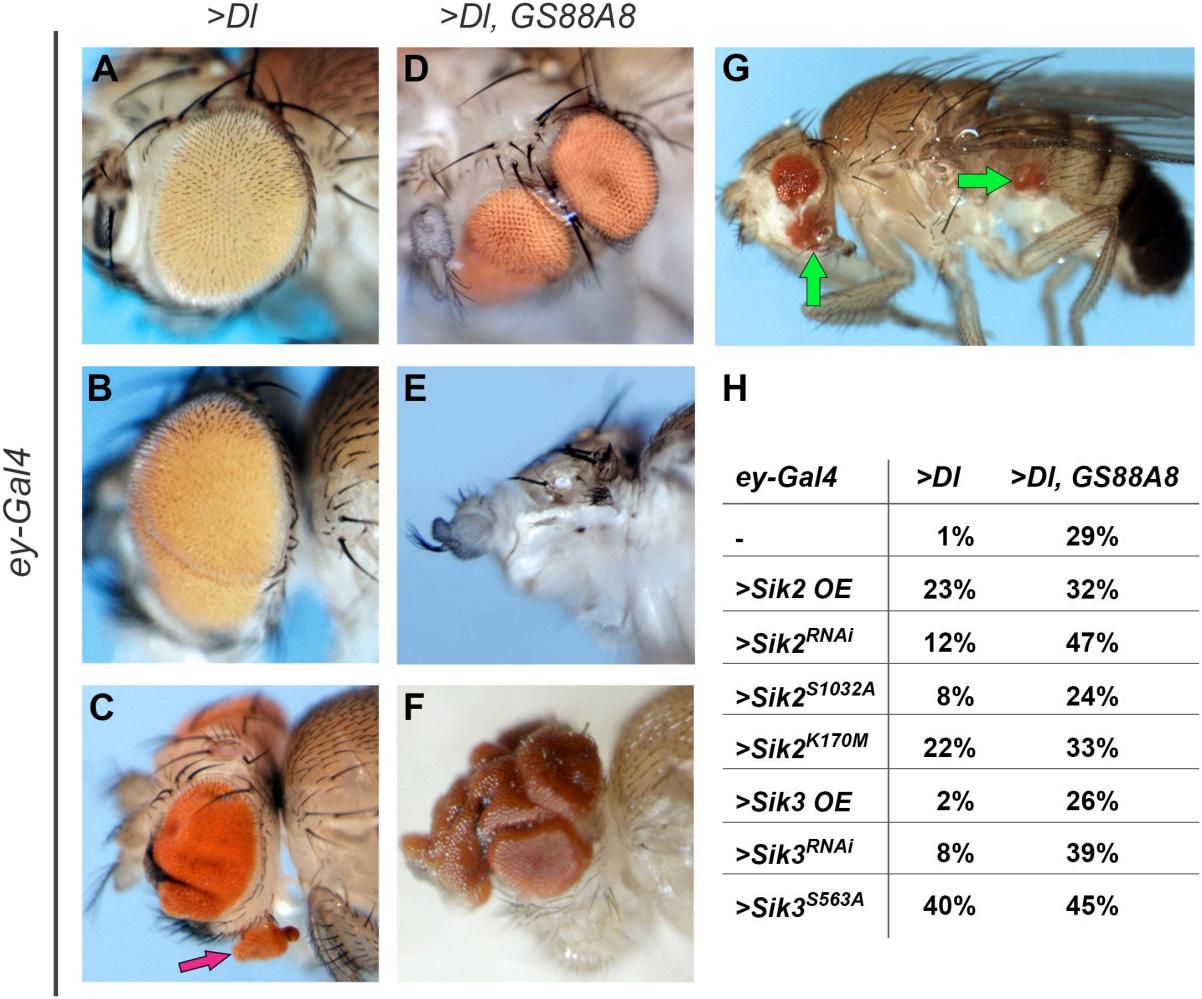
The tumor-like effect of salt inducible kinases (Siks). (A-G) Examples of “sensitized” and “eyeful” crosses’ progeny. (A-C) Examples of flies chosen from the sensitized background (*ey > Dl*) (A) Representative fly from sensitized background showing slight eye overgrowth, accepted as baseline. (B) An example of eye overgrowth, accepted as affected. (C) An example of ectopic eye formation, indicated with a pink arrow. (D-G) Examples of flies chosen from the eyeful background (*ey > Dl*, *GS88A8*) (D) An example of split eyes; an eye was split into two on one side of the head. (E) An example of loss of differentiated eye tissue and enlarged antenna. (F) An example of excessive eye growth. (G) A representative example of metastasis. The green arrows indicate proximal and distal metastasis of eye tissue. (H) Sensitized (*ey > Dl*) and eyeful (*ey > Dl*, *GS88A8*) flies were crossed with the Sik transgenes: background, crossed with wild type *w*^*1118*^ (-), Sik2 overexpression (>*Sik2* OE), Sik2 knock-down (>*Sik2*^*RNAi*^), Sik2 constitutively active (>*Sik2*^*S1032A*^), Sik2 kinase-dead (>*Sik2*^*K170M*^), Sik3 overexpression (>*Sik3* OE), Sik3 knock-down (>*Sik3*^*RNAi*^), and Sik3 constitutively active (>*Sik3*^*S563A*^). For each cross, normal eyes (similar to the sensitized background, like in (A)), and affected eyes (eye folding or overgrowth, split eyes or ectopic eye tissue, eye loss or antenna enlargement, and metastasis, like in (B-G)), in the progeny were quantified. The ratio of cumulative number of affected eyes over the total eye number is presented in the table H. The detailed analysis is shown in S1 Table. The full genotypes are listed in S2 Table. Anterior is to the left, ventral is down.

Our previous experiments in mammalian cell culture indicated that Sik proteins are involved in cancer progression [36]. Thus, we addressed whether they can have similar effects *in vivo* and employed “eyeful”, a *Drosophila* Notch pathway-dependent eye tumor model. Overexpression of the Notch ligand Delta using the *ey-GAL4* driver, which expresses a UAS target specifically in embryonic eye primordia and in the third instar larval stage in cells after the morphogenetic furrow (MF) (S1C Fig) [3], induces eye overgrowth and sensitizes the organ to tumorigenesis (Fig 1A) [4, 54]. “Eyeful” flies, which overexpress Delta and concurrently carry the *GS88A8* mutation (deregulation of the epigenetic silencers *lola* (*CG12052*) and *psq* (*CG2368*)), display further eye enlargement and tumorigenesis [2, 54]. We tested Sik function both in the “sensitized” (*ey>Delta*) and “eyeful” (*ey>Delta*, *GS88A8*) backgrounds by crossing in different Sik transgenes.

All tested genetic combinations affected eye development to some extent. Affected eyes showed different phenotypes including tumor-like overgrowth, incidences of small ectopic eyes or full-size but split eyes, total loss of eye tissue, or metastasis that originated from the dissemination of transformed cells from the developing eye to the body (Fig 1A–1G). For each genetic combination, the ratio of affected eyes was quantified by analyzing and counting each eye independently. Eyes that resembled the parents were not considered as affected; any deviation from the most common baseline phenotype was considered to be affected. In the sensitized background, Sik2 overexpression (*Sik2* OE) affected 23% of the eyes, Sik2 knock-down (*Sik2*^*RNAi*^) 12% of the eyes, Sik2 constitutively active (*Sik2*^*S1032A*^) affected 8% of the eyes, and Sik2 kinase-dead (*Sik2*^*K170M*^) affected 22% of the eyes. While Sik3 overexpression (*Sik3* OE) did not cause any obvious eye phenotype when compared to the baseline, Sik3 knock-down (*Sik3*^*RNAi*^) affected 8% of eyes (Fig 1A–1C and 1H, S1 Table). The true eye tumor model (eyeful) results were difficult to interpret, since the genetic background alone already caused a high ratio of affected eyes; yet they were not contradictory to the ones obtained with the “sensitized” background (Fig 1D–1F and 1H, S1 Table). Taken together, our results show that any modulation of Sik activity (both increases and decreases) enhanced both tumor-like phenotypes and eye loss phenotypes, possibly indicating a disturbance in eye development. Interestingly, the wild type overexpression of Sik2 (*Sik2 OE*) and misexpression of the kinase inactive form of Sik2 (*Sik2*^*K170M*^) led to similar phenotypes rather than opposite ones. This prevented us from classifying Sik2 as a tumor suppressor or oncogene. The observation that overexpression of wild type Sik3 (*Sik3 OE*) and knock-down of Sik3 (*Sik3*^*RNAi*^) had a milder effect than manipulating Sik2 would seem to suggest that Sik3 might not be involved in this process as much as Sik2; however constitutively active Sik3 (*Sik3*^*S563A*^, which cannot be suppressed by cAMP signaling), caused the most pronounced phenotypes of all. *Sik3*^*S563A*^ was mostly lethal both in the sensitized and eyeful backgrounds, which might be due to ectopic expression of *ey-GAL4* [55]. The few escapers that we were able to analyze displayed a high percentage of affected eyes (40% in the sensitized and 45% in eyeful backgrounds); also, severe tissue defects, including differentiation problems of eye and antenna growth (Fig 1E) and rare instances of distal metastasis of eye tissue to the body (Fig 1G) were detected. The observed eye loss phenotypes suggest a possible role for Sik in retinal fate determination.

Taken together, depletion or overexpression of Siks enhanced tumor-like growth in the eye in the sensitized and eyeful backgrounds. Constitutively active Sik3 may have additional effects, as can also be seen below (Fig 3).

### Siks have a role in the developing eye

To understand if Siks are required for normal patterning of the eye disc, Sik activity was specifically altered via two strong drivers. In this experimental set-up, in addition to the *ey-GAL4* driver described in the previous experiment, we introduced *lGMR-GAL4* to drive expression in all PRs during larval stages and throughout adulthood [56], and *UAS-Dicer2* to amplify the RNAi effect.

**Fig 2:**
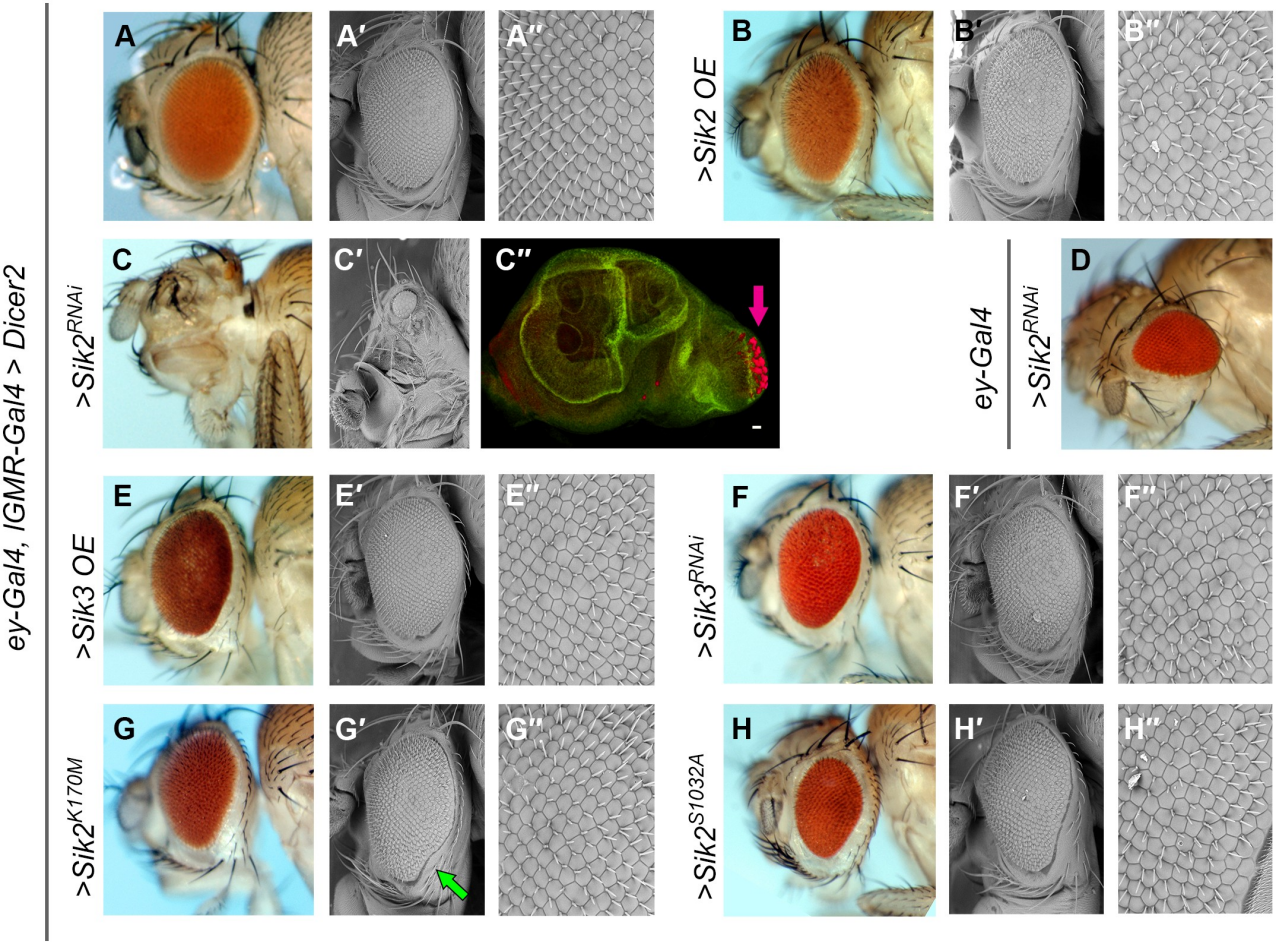
Eye morphology changes after manipulating Sik activity in the developing retina. Images in A, B, C, D, E, F, G, H represent light microscopy, A′, A′′, B′, B′′, C′, E′, E′′, F′, F′′, G′, G′′, H′, H′′ scanning electron microscopy, C′′, confocal microscopy images. In all cases except D, the flies are in “*UAS-Dicer2 / +; ey-GAL4*, *lGMR-GAL4 / +*” background. (A-A′′) Control (driver crossed with wild type, *w*^*1118*^). (B-B′′) Sik2 overexpression (>*Sik2* OE). (C-C′′) Sik2 knock-down (>*Sik2*^*RNAi*^) (C′′) Third instar larval eye disc. Red is elav (neurogenic), pink arrow indicates the MF, green is Delta (non-specific stain, background signal). Scale bar 20 μm. (D) Sik2 knock-down by *ey*-GAL4 driver and two copies of *UAS-Sik2*^*RNAi*^. (E-E′′) Sik3 overexpression (>*Sik3* OE). (F-F′′) Sik3 knock-down (>*Sik3*^*RNAi*^). (G-G′′) Sik2 kinase-dead (>*SIK2*^*K170M*^). The green arrow indicates the ventral notch. (H-H′′) Sik2 constitutively active (>*Sik2*^*S1032A*^). The full genotypes are listed in S2 Table. In all pictures, anterior is to the left, ventral is down.

Modulating Sik protein levels with eye-specific drivers disrupted the structural integrity of the eye. Overexpression of wild type Sik2 (*Sik2* OE) and Sik3 (*Sik3* OE) or Sik2 point mutants (*Sik2*^*K170M*^ and *Sik2*^*S1032A*^), as well as Sik3 knock-down (*Sik3*^*RNAi*^), caused mild defects in the organization of ommatidia, cone cells, lens structure, and resulted in supernumerary bristles as shown by light microscopy and scanning electron microscopy (Fig 2A–2B′′ and 2E–2H′′).

Sik2 knock-down (*Sik2*^*RNAi*^) not only affected the cellular organization (intermediate knock-down, Fig 2D), but also drastically decreased eye size with full penetrance (strong knock-down, Fig 2C). Furthermore, it resulted in enlargement of the antenna in some instances, a phenotype also visible in eye-antennal imaginal discs (Fig 2C′′). This extreme phenotype could be attributed to off-target effects of the *Sik2*^*RNAi*^ line, which was shown to additionally downregulate Sik3 by a previous report [30]. The opposite phenotype of the *Sik2*^*RNAi*^ line, overgrowth, seen in the previous experiment (Fig 1) might be attributable to the fact that the eye tissue was already primed for growth by Delta overexpression. Overall, these results suggest that Sik2 and Sik3 activity may be necessary for proper eye development.

Mutant alleles are available for both Sik2 and Sik3. Surprisingly, the Sik2 mutant (*Sik2*^*Δ41*^) did not exhibit lethality or any obvious defect in the eye, although the lens and bristle defects were not examined by electron microscopy. Sik3 null mutants (*Sik3*^*Δ109*^) are lethal at an early stage, most probably due to a metabolic problem [57]. Thus, we investigated *Sik3* null mutant eye tissue by generating eye-specific mitotic clones. We created *Sik3* null mutant clones at the larval stage both in the entire eye, selecting against the apoptotic gene *hid* (S3A′′ Fig), and in eye clones that were marked by absence of the *white* gene (S3B′ Fig). Loss of Sik3 in clonal regions did not visibly affect the eye structure or cellular organization in either experiment (S3A′′ and S3B′ Fig), although it was not examined by electron microscopy. Taken together with the results obtained with the *Sik2*^*RNAi*^ line (Fig 2C–2C′′), compensation between the two Sik homologs appears to be likely.

Ectopic expression of a kinase-dead form of Sik2 (*Sik2*^*K170M*^) or a constitutively active form of Sik2 (*Sik2*^*S1032A*^) not only resulted in cone and bristle cell defects, but also a ventral growth defect, a “notch” on the ventral side of the eye (Fig 2G–2H′′, indicated with a green arrow) that is not present in controls (Fig 2A–2A′′). The phenotype obtained by intermediate knock-down of Sik2 resembles loss of the ventral half of the eye (Fig 2D), although this was not confirmed by loss of ventral eye markers at the larval level. These ventral eye phenotypes were seen with point mutation alleles of Sik2 but not with Sik3, suggesting that Sik2 can more potently affect ventral eye development than Sik3.

Ventral eye defects can be caused by misregulation of dorsal-ventral (D-V) patterning during development. It is long known that D-V polarity is established at early larval stages by the activation of the Notch receptor in the midline cells of the eye disc by Delta and Serrate from dorsal and ventral sides respectively [4] (S1B Fig). Our data indicate that disrupting the Sik balance results in phenotypes that resemble Notch pathway defects, suggesting a possible interaction of Siks with the Notch signaling pathway.

### Siks interact with the Notch pathway

Our manipulations of Siks in the eyeful and sensitized backgrounds, and during normal eye development, indicate a possible interaction with the Notch pathway. To better understand this interaction, we used the driver *ey-GAL4*, *lGMR-GAL4* with UAS-Dicer2 to overexpress or deplete Notch pathway components in combination with *Sik* transgenes.

**Fig 3:**
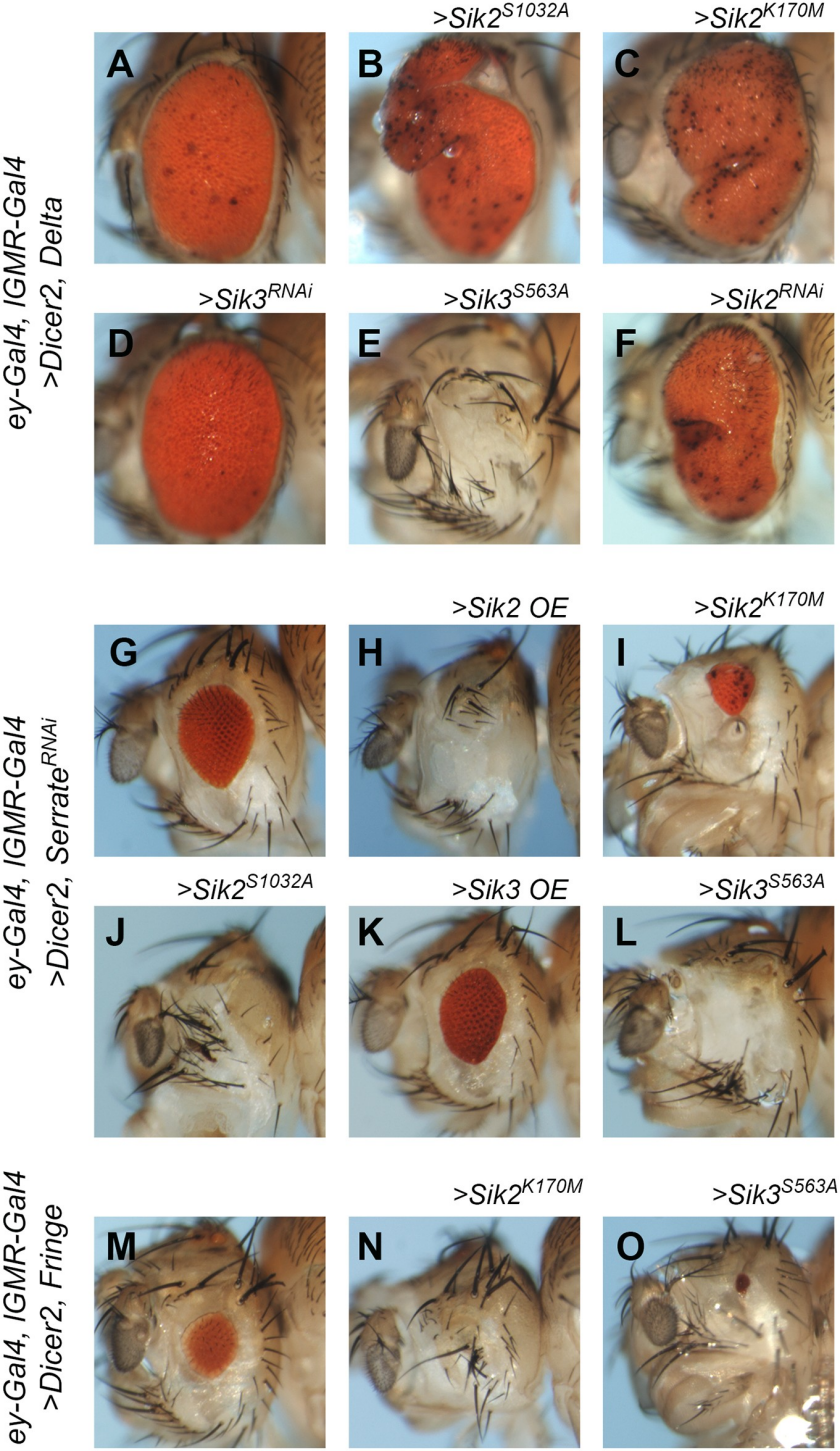
Genetic interaction between sik transgenes and Notch pathway members. (A-F) Delta overexpression with strong eye specific driver (*UAS-Dicer2 / UAS-Delta; ey-GAL4*, *lGMR-GAL4 / +*) in combination with overexpression of *Sik* transgenes and RNAi depletion. (A) Control. (B) Sik2 constitutively active (>*Sik2*^*S1032A*^); example of overgrowth and necrosis. (C) Sik2 kinase-dead (>*Sik2*^*K170M*^); example of overgrowth and necrosis. (D) Sik3 knock-down (>*Sik3*^*RNAi*^). (E) Sik3 constitutively active (>*Sik3*^*S563A*^); example of total eye loss. (F) Sik2 knock-down (>*Sik2*^*RNAi*^). (G-L) Serrate knock-down with strong eye specific driver (*UAS-Dicer2 / +; ey-GAL4*, *lGMR-GAL4 / +; Serrate*^*RNAi*^ */ +*) in combination with overexpression of Sik transgenes. (G) Control. (H) Sik2 overexpression (>*Sik2* OE). (I) Sik2 kinase-dead (>*Sik2*^*K170M*^). (J) Sik2 constitutively active (>*Sik2*^*S1032A*^). (K) Sik3 overexpression (>*Sik3* OE). (L) Sik3 constitutively active (>*Sik3*^*S563A*^). (M-O) Fringe overexpression by strong eye specific driver (*UAS-Dicer2 / +; ey-GAL4*, *lGMR-GAL4*, *UAS-Fringe / +*) in combination with overexpression of Sik transgenes. (M) Control. (N) Sik2 kinase-dead (>*Sik2*^*K170M*^). (O) Sik3 constitutively active (>*Sik3*^*S563A*^). All controls are crossed with wild type, *w*^*1118*^. The full genotypes are listed in S2 Table. In all pictures, anterior is to the left, ventral is down.

We first tested the Notch ligand Delta, which is dorsally expressed in the late L2 stage to establish D-V polarity, and also expressed at late L3, immediately posterior to the MF, to determine the number of neurons specified from the epithelium (S1B and S1C Fig). As in the “sensitized” background (Fig 1A), overexpression of Delta resulted in larger eyes (Fig 3A). This phenotype was enhanced by adding the constitutively active (*Sik2*^*S1032A*^) or kinase-dead (*Sik2*^*K170M*^) forms of Sik2, which caused tissue folding and necrosis (Fig 3B and 3C) or in some cases, a total loss of the eye. Comparing these phenotypes to the Sik2 point mutation transgenes in the same background without coexpression of Delta, which produced a minor effect on eye size that is limited to the ventral eye (Fig 2G and 2H), these results clearly indicate a genetic interaction between Sik2 and Delta. While Sik3 knock-down (*Sik3*^*RNAi*^) did not alter the phenotype (Fig 3D), constitutively active Sik3 (*Sik3*^*S563A*^), which caused excessive defects in the “sensitized” and “eyeful” backgrounds, resulted in phenotypes that are similar to those obtained with constitutively active and kinase-dead Sik2 lines, including both larger eyes with necrotic lenses and total eye loss (Fig 3E). Sik2 knock-down (*Sik2*^*RNAi*^) in combination with Delta overexpression led to a phenotype very similar to those obtained with Sik2 point mutations; larger eyes and necrotic lenses (Fig 3F). Keeping in mind that Sik2 knock-down itself (*Sik2*^*RNAi*^) in the same background prominently blocked eye growth with full penetrance (Fig 2C), we can conclude that coexpression of Delta rescued the Sik2 knock-down phenotype. This experiment suggests that Delta acts downstream of Siks and thus, Siks might be regulating Delta or its main effectors.

Serrate is another Notch ligand, and is expressed in the ventral half of the eye imaginal disc. Depletion of Serrate using *Serrate*^*RNAi*^ resulted in small eyes (Fig 3G). Sik2 overexpression (*Sik2* OE) in a Serrate-depleted background enhanced the small eye phenotype and caused total loss of the eye in many instances (Fig 3H). Similar phenotypes were observed when Sik2 kinase-dead (*Sik2*^*K170M*^) or constitutively active Sik2 (*Sik2*^*S1032A*^) were used (Fig 3I and 3J), suggesting a genetic interaction between Sik2 and Serrate, which we confirmed using another *Serrate*^*RNAi*^ line. Sik3 overexpression (*Sik3* OE) (Fig 3K) or Sik3 knock-down did not have any noticeable effect when combined with *Serrate*^*RNAi*^, just like in the Delta overexpression background (Fig 3D). On the other hand, the Sik3 constitutively active form (*Sik3*^*S563A*^) enhanced the small eye phenotype and resulted in total loss of the eye (Fig 3L). The constitutively active Sik3 phenotype was exaggerated as in Figs 1 and 3E, which more closely resembled the phenotypes observed with Sik2 rather than wild type Sik3.

The glycosyltransferase Fringe is expressed in the ventral eye disc. It modifies Notch to promote Notch-Delta interaction, and suppresses interaction of Notch with Serrate except for at the dorsal-ventral border (S1B Fig). As previously reported, *Fringe* overexpression results in smaller eyes, presumably by disrupting the balance of Notch activation during development (Fig 3M) [58]. The small eye phenotype of Fringe was not altered by Sik3 overexpression, but it was greatly enhanced when combined with the kinase-dead form of Sik2 (*Sik2*^*K170M*^) or the constitutively active version of Sik3 (*Sik3*^*S563A*^) (Fig 3N and 3O), indicating a genetic interaction between Siks and Fringe.

Taken together, these observations reveal a genetic interaction between Notch pathway members and Siks, in particular Sik2. Since Sik2 is a protein kinase, we hypothesized that it targets and phosphorylates members of the Notch pathway, or affects Notch signaling indirectly through the effectors of the Notch signaling.

In order to investigate if Notch pathway members could be directly targeted by Siks, we set out to find Sik phosphorylation motifs on Notch pathway members. First, we aimed to determine the amino acid sequence targeted by Siks. In addition to previously published Sik target motifs, recently identified targets were included in this study, to generate an updated target sequence. For this purpose, first, we aligned and compared experimentally-deduced Sik target sequences to generate an updated Sik target sequence (S4A Fig). Since only a few Sik targets are experimentally confirmed in *Drosophila*, we made use of proven targets from different organisms. This gave us a more generic, but a more conserved sequence of amino acids. SCARB1 and SREBP-1 were excluded from this analysis, since they do not contain any of the conserved residues. It is therefore possible that Siks have a second selection mechanism for phosphorylation. In particular, a 15 amino acid long region flanking the serine / threonine residue that is phosphorylated by Sik in different species was aligned to search for a target motif (S4A Fig). An amino acid sequence with 80% consensus among different targets was accepted as the target motif: [L/W]X[R/K]XX[S/T]*XXXL (* marks the serine / threonine residue phosphorylated by Sik) (S4B Fig). After determining the target motif, we scanned the *Drosophila melanogaster* proteome for proteins carrying this motif. In the whole fly proteome, this motif was identified in 307 annotated proteins, which include Notch (on serine 1066), Delta (on serine 740), and Fringe (on threonine 303), along with previously identified and confirmed targets (S4C Fig, S3 Table). However, the predicted residues are located in the non-cytosolic portions of these transmembrane proteins. Since Siks are known to function at nuclear or cytoplasmic compartments, it seems unlikely that these residues are phosphorylated by Sik proteins directly.

### Siks are expressed in the developing retina

Our observations suggest a role for Siks in eye development, implying that Siks must be expressed in the developing retina. Thus, we set out to determine the localization of these proteins. Due to a high conservation of AMPK family members, most antibodies have the potential problem of cross-reactivity. To confirm their specificity, several commercially available antibodies were first tested in western blot (WB) analyses on crude fly extracts and immunofluorescence (IF) stainings on dissected larval and sectioned adult tissues. None of the commercially available α-SIK2 antibodies were able to specifically detect fly Sik2 in WB or IF. Sik3 has two known isoforms. The long isoform has previously been shown to interact with the Salvador (Sav) protein [30]. However, using the Abcam ab88495 α-human SIK3 antibody in WB, we observed predominantly the Sik3 short isoform. This antibody recognizes fly Sik3 at >75 kD (S2B Fig), slightly higher than the calculated size (77 kD), which was also confirmed by overexpression of Sik3. While we were able to show that *Drosophila* Sik3 is highly expressed in the developing larval nervous system in WB analyses (S2B Fig), no specific staining for Sik3 could be obtained in IF analyses using the same antibody.

Since none of the tested antibodies were specific enough to recognize and distinguish fly Sik2 and Sik3 in IF experiments, in order to observe endogenous protein localization, we generated BAC transgenic constructs in which Sik2 and Sik3 were tagged with fluorescent proteins. BAC clones spanning the entire coding sequence, UTRs and 14 and 6 kb upstream regulatory region of the *Sik2* and *Sik3* genes respectively were used; green fluorescent protein (Sik2) or mCherry (Sik3) sequences were inserted C-terminally, prior to the stop codon, using the P[acman] recombineering technique [41] (S2A Fig).

Eye imaginal discs of BAC transgenic flies were co-stained for cell-type specific markers and for GFP or mCherry to identify the localization of Sik2 and Sik3 proteins, respectively. Senseless (Sens) was used as a marker for PR R8 [59], Prospero (Pros) as a marker for R7 and cone cells [60], Spalt-major (Salm) as a marker for R3/R4 cells for the initial few rows posterior to the MF and R7/R8 for more posterior cells [61], Seven-up (Svp) was used as a marker for R3 and R4 (expressed stronger in R4 than in R3) and R1 and R6 [62], and Rough (ro) as a marker for PRs R2/R5 [59]. Additionally, N-Cadherin (N-Cad) and Armadillo (Arm) were used to mark the AJs. They are known to localize to the apical surface, between cone cells and PRs in pupal retinae [63, 64].

**Fig 4:**
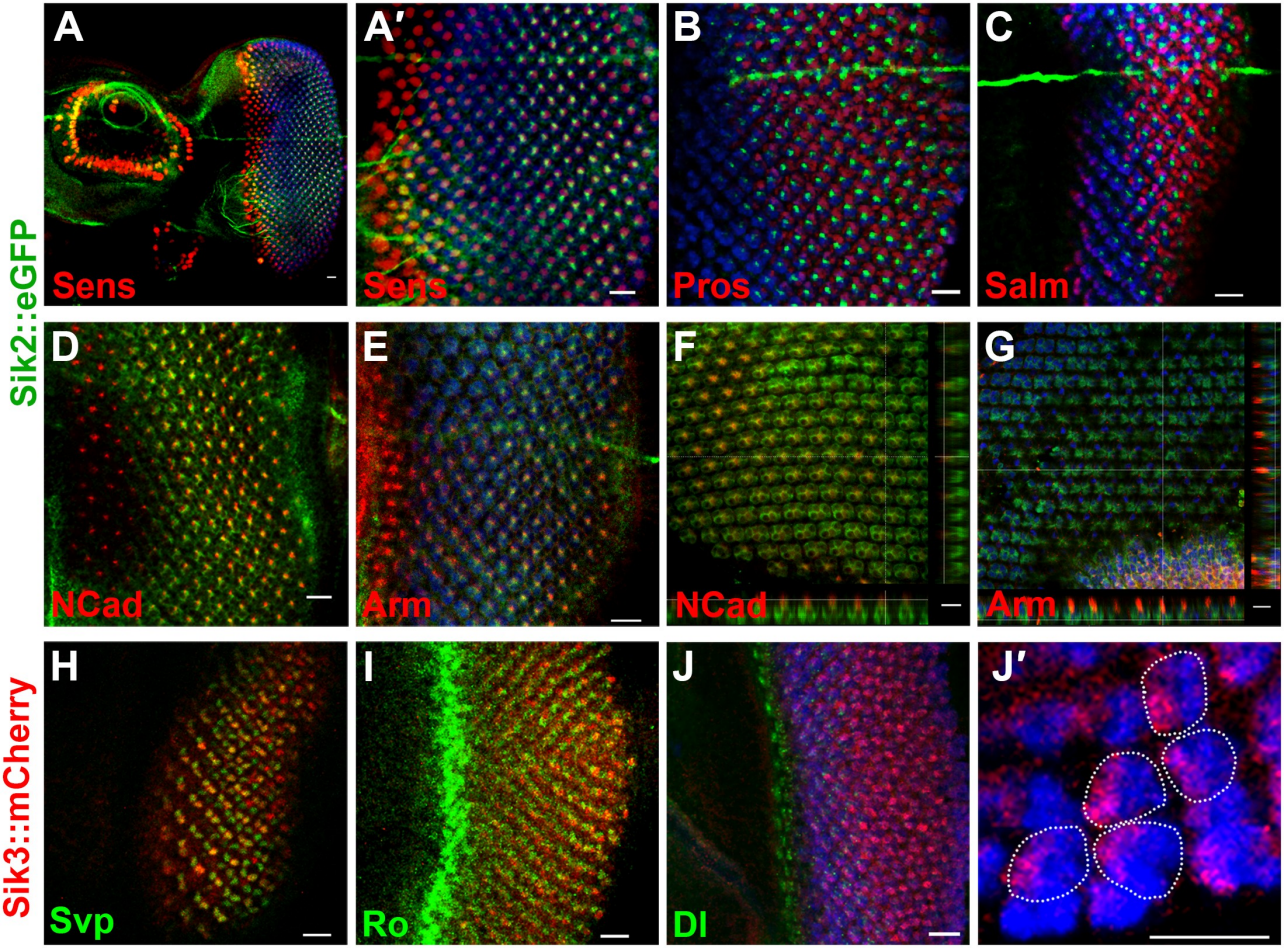
Expression profile of Sik2 and Sik3 in the developing retina. A, A′ and J are maximum projections; the rest of the acquisitions are single sections. A, A′, B, C, D, E, H, I, J, J′ are 3^rd^ instar larval eye disc. F, G are ~30% pupal retinae. Wherever used, Elav appears in blue, marking neural cells. (A-G) Sik2::eGFP transgenics. Sik2 is shown in green (A-A′), Senseless (Sens) is shown in red, marking the photoreceptor R8. (A′) Inset of (A. B) Prospero (Pros) shown in red, marking R7 and cone cells. (C) Spalt-major (Salm) shown in red, marking R3 and R4 in ommatidia immediately posterior to the morphogenetic furrow (MF) and R7 and R8 in more posterior ommatidia. (D) N-Cadherin (N-Cad) shown in red, marking Adherens Junctions (AJs) at larval stage. (E) Armadillo (Arm) shown in red, marking AJs at larval stage. (F) N-Cad shown in red, marking AJs at pupal stage. (G) Arm shown in red, marking AJs at pupal stage. (H-J′) Sik3::mCherry transgenics. Sik3 shown in red (H) Seven-up (Svp) shown in green, marking R3/R4 in early ommatidia and R1/R6 in ommatidia more posterior. Sample is mid-3^rd^ instar larva. (I) Rough (ro) shown in green, marking R2/R5 immediately posterior to the MF. (J) Delta (Dl), the neurogenic factor shown in green. (J′) Inset of J, Dl signal is not represented here. The ommatidium borders are marked with dashed line. Posterior to the right and scale bar is 10 μm in all stainings.

In late 3^rd^ instar larval eye discs, a Sik2::eGFP signal was observed in the center of every ommatidium, starting 6–7 rows posterior to the MF (Fig 4A–4E). The localization does not appear to be nuclear, thus no co-localization was observed with Sens (Fig 4A and 4A′), Pros (Fig 4B), or Salm (Fig 4C), all central PR markers that are localized to the nucleus [59–61]. Sik2::eGFP was observed to be close to the N-Cad and Arm signals at the larval stage (Fig 4D and 4E), though the Sik2 signal did not exactly co-localize with the junctional proteins. At ~30% pupal development, the Sik2 signal in the PRs was localized basal to the AJ proteins Arm and N-Cad, and dispersed in the cytoplasm (Fig 4F and 4G).

In the L3 eye discs, mCherry tagged Sik3 appears to be nuclear. Co-staining of Sik3::mCherry with Svp showed a co-localization with photoreceptors R3/R4 starting at row 3 (Fig 4H), and not with Ro (Fig 4I). Delta (Dl) was expressed both in Sik3-positive and Sik3-negative cells in the initial few rows of ommatidia (Fig 4J). In the anterior rows, Sik3 was clearly expressed in the anterior-most two PRs of every ommatidium, which represent the position of R3/R4 cells (Fig 4J′). Posterior to this, Sik3 was expressed in most PR cells (Fig 4J).

In summary, Sik2 appears as a cytoplasmic, PR-specific protein, localizing basal to cell junctions; and Sik3 appears as an R3/R4-specific protein initially, that is later expressed pan-neurally during larval retina development.

## Discussion

Precise regulation of signaling pathways and the interactions between them is required for proper tissue development. One of the fundamental aims of developmental biology is to identify the genetic interaction network that directs the development of a tissue. Although we are far from understanding the complete picture, dissecting the role of major interactors will get us closer to the answer. In this study, we focused on fly homologs of Sik2 and Sik3, their importance for development of the retina, and their involvement in tumor progression. *Sik* genes are members of the AMPK family that are conserved in animals and plants. They function as regulators of sodium sensing, cAMP signaling, metabolism, bone formation, immune cell maturation and neuroprotection (reviewed in [65]). Although some Sik functions like gluconeogenesis and lipogenesis have been thoroughly studied, knowledge about their involvement in neural development is quite limited.

Molecular analyses revealed that development and tumor progression actually share many aspects; there are fundamental molecular mechanisms common to both processes [2]. The *Drosophila* eye has been used as model to investigate developmental processes for decades. Lately, it has also been employed in tumorigenesis studies (reviewed in [66]). The eye undergoing metamorphosis is a great tool to study both the genetic interactions that control *de novo* tissue generation, and the molecular mechanisms that control cell division to establish proper tissue size. With this motivation, we tested Sik behavior in an insect tumor model.

Although well-known for its developmental role, Notch pathway activity was also shown to be disturbed in several cancer types, acting as tumor suppressor or oncogene in a context-dependent manner (reviewed in [67, 68]). Our first experiment tested whether Siks could modulate the phenotype of a Notch-based tumor model in the *Drosophila* eye. We first tested in the context of gain of function of the Notch ligand Delta, which alone causes slight overgrowth and sensitization of the tissue to tumorigenesis, and then in the “eyeful” tumor model. While the contribution of Siks was less obvious in the stronger “eyeful” background, in the “sensitized” background changes in Sik2 levels clearly induce tumor-like structures (Fig 1, S1 Table). Paradoxically, our data show that both gain and loss of function of Siks result in similar phenotypes, preventing us from easily classifying Siks as inducers or inhibitors of growth.

The misregulation of Sik2 and Sik3 being detected in several cancer cases [39, 48] at both genetic and epigenetic levels [69], and Sik3 being established as an ovary cancer marker [70] are consistent with our finding that Siks can promote tumorigenesis. In addition to the previous correlation studies, our experiment shows that Sik misregulation can be causative for tumor formation. Siks have been associated with certain cancer types, with both oncogenic and tumor suppressor roles, suggesting Sik involvement at a very fundamental level in cancer-related mechanisms. This dual role is reminiscent of Notch-mediated neoplasias, where Notch acts either as an oncogene or tumor suppressor gene depending on the cellular context [71].

As demonstrated in Fig 1, Siks contribute to tumorigenesis and metastasis. It is known that reduction in E-Cadherin and destabilization of AJs is a key step for the epithelial-mesenchymal transition that is a prerequisite for metastasis [72, 73], and any compromise in AJ stability is correlated with an invasive phenotype. The localization of Sik2 around the cell junctions (Fig 4D–4G) appears to be in line with this finding. We have previously shown that Sik alters the invasiveness of tumor cells [36], and other reports propose Siks as anti-metastatic proteins [74, 75] and show that loss of Sik function disables anoikis [76]. Combining the ability of Sik to contribute to invasiveness with their effect on survival, Siks are strong candidates for promoting cancer and anoikis.

The structural analysis by SEM of gain and loss of function alleles of Sik2 and Sik3 suggest that Sik proteins may play a role in the proper development of the eye. We observed that misregulation of Siks resulted in defects, however no obvious phenotypes were observed in mutants of *Sik2* or *Sik3* (S3 Fig). This discrepancy in our results might be attributed to functional compensation mechanisms that are employed in the absence of one of the Sik homologs, either with one another, or with other members of the AMPK family. The severe defects observed with the *Sik2*^*RNAi*^ line (Fig 2C and 2D), which is known to downregulate Sik3 as well as Sik2 [30], supports this idea, as loss of both homologs is expected to have a more severe phenotype than loss of either one alone.

It is intriguing that overexpression of a kinase-dead form of Sik2 (*Sik2*^*K170M*^) perturbed eye development and promoted tumorigenesis in a similar way to the wild type Sik2 protein (Figs 1, 2 and 3). This implies that the phenotypes are not wholly the result of aberrant phosphorylation of direct Sik targets. Sik2^K170M^ could presumably retain molecular interactions with its targets, without phosphorylating them, and possibly with other interactors such as scaffold proteins. This might result in an unforeseen dominant negative effect, effectively creating another Sik2 loss of function condition in addition to knock-out (full mutant) and knock-down (RNAi). The constitutively active transgenes, which are not subject to PKA suppression, represent gain of function alleles of Siks that would be expected to give stronger phenotypes than the wild type overexpression lines. Constitutively active Sik2 (*Sik2*^*S1032A*^) expressed in the eye gives similar results to wild type overexpression (Fig 2), lending further support to the hypothesis that these phenotypes are not wholly dependent on the kinase activity of Sik2.

Gain and loss of function of Sik3 via overexpression and knock-down resulted in defects in lens and bristle cells that were similar to each other, and to the Sik2 manipulations (Fig 2E–2F′′), perhaps indicating that Sik3 also has a role in eye development. Another surprise was the different effect of Sik3 knock-down by RNAi (disorganization of ommatidial packing and some supernumerary bristles) compared to Sik3 null mutant clones. This might be explained by the *Sik3*^*RNAi*^ line having off-target effects and affecting other homologs.

The effect of overexpressing constitutively active Sik3 (*Sik3*^*S563A*^) was stronger than the overexpression of wild type Sik3 in multiple backgrounds (Figs 1H and 3E, 3L and 3O). The difference was much more pronounced than the difference between wild type and constitutively active Sik2. One possible explanation is that PKA phosphorylation is more important for normal regulation of Sik3 activity than Sik2, which might be restrained by additional mechanisms. Alternatively, we may have disrupted the balance of AMPK family members by overexpressing the constitutively active Sik3 protein.

Overexpression of Sik2^K170M^ or Sik2^S1032A^ did not prevent the generation of eye tissue, but did induce a growth defect in the ventral eye (Fig 2G–2G′′ and 2H-2H′′). This highly resembles Notch pathway phenotypes [77] (reviewed in [78]). The observation of flies that appear to be missing the entire ventral eye following intermediate knock-down of Sik2 (Fig 2D) further suggests that Siks, particularly Sik2, are able to influence dorsoventral polarization, or growth or survival of ventral cells, in the developing eye tissue in a Notch-dependent manner. Our results imply that Sik can affect the activity of the Notch pathway, possibly by interacting with Notch pathway components or their effectors. However, the exact contribution of Siks in this process still needs to be established.

Having observed that Siks modify the Delta overexpressing background, and that manipulation of Siks can affect eye development in ways reminiscent of Notch pathway defects, we sought further evidence of genetic interactions by co-overexpressing Siks with Notch pathway components in the eye (Fig 3). We found that manipulation of Sik2 levels exaggerate the small eye phenotype of Serrate knock-down (Fig 3G–3J) and of Fringe overexpression (Fig 3M and 3N), although they do not affect eye size on their own (Fig 2B–2B′′ and 2G–2H′′). Unlike Fringe overexpression and Serrate knock-down, Delta overexpression increases eye size, since it is not only required for establishment of D-V polarity at the L2 stage, but also promotes neurogenesis behind the MF in the L3 stage [79]. Changes in Sik2 levels also exaggerate or modify this phenotype (Fig 3A–3C and 3F). Conversely, while Sik2 knock-down strongly suppresses eye growth (Fig 2C), Delta overexpression can rescue this phenotype (Fig 3F), suggesting that Delta acts downstream of Sik2. Therefore, although we have not verified a direct interaction between Siks and Notch pathway members, our data clearly show that they are epistatic to each other.

After confirming a genetic interaction between Siks and Notch, we asked whether Notch pathway members might be direct targets of Sik phosphorylation. By comparing the sequences of experimentally proven Sik targets, we identified a consensus phosphorylation motif (S4A and S4B Fig). Analysis of the fly proteome showed that >300 reviewed protein entries contain this deduced motif; the list includes Notch, Delta and Fringe from the Notch pathway [80]. Notch and its ligands are known to be regulated by phosphorylation of the intracellular parts of the proteins (reviewed in [81]). However, the motifs predicted to be phosphorylated by Siks are located in the extracellular regions of Notch and Delta. In the case of Fringe, the predicted Sik target motif would be located in the Golgi lumen. Since the Sik proteins function in the cytosol, these sequences cannot represent true Sik targets. We thus hypothesize that Siks might be modulating the Notch signaling pathway indirectly, maybe by phosphorylating a regulator of Notch or its ligands, or a major downstream effector.

Scanning the fly proteome revealed experimentally-confirmed Sik targets like Salvador, showing the validity of our screening. Novel predicted targets include several DNA and RNA binding proteins, transcription coactivators, proteins important for the RNAi machinery, epigenetic regulators, some proteins important in cell cycle and cancer, proteins related to cytoskeletal organization, proteins functioning in signal transduction, some proteins functioning in phototransduction, gustatory and odorant receptors, several ubiquitin-protein ligases, some proteins important in lipid synthesis, many mitochondrial proteins, and proteins with metallopeptidase activity that are important for migration (S3 Table). Our data hint that several proteins related to animal development and retina morphogenesis that could be Sik targets, including UVRAG from the Notch signaling pathway, Pannier, Slingshot, tiptop, hedgehog, hopscotch, Calpain-D, Neurobeachin, fork head, prospero, FGFR2, flamingo, elav, Dscam2, Patj, scribble, arrowhead and domeless. It will be interesting to explore some of these hits further in future experiments. Pannier is particularly interesting, since it is responsible for establishing the dorsal eye fate, and we show that Sik disruption leads to a ventral eye defect. Clearly, the predicted putative phosphorylation sites (S3 Table) need biochemical confirmation.

Recent findings point to a potential role of Sik kinases in animal development, mostly in the skeletal tissue [82]. We aimed to investigate their role in development, but were hampered by the lack of specific fly antibodies. To overcome this, we generated novel useful tools. Using the BAC recombineering technique, we created translational fusion proteins of Sik2 and Sik3 with fluorescent protein tags (S2A Fig). With the only working α-SIK3 antibody, our WB analyses showed that Sik3 was highly expressed in the larval nervous system (brain, ventral nerve cord and eye-antennal imaginal discs) (S2B Fig), unlike the ubiquitous expression shown previously [83]. In contrast to a previous report [30], we mainly detected the shorter isoform of Sik3 (S2B Fig) and thus used the Sik3-RA transcript to generate our fluorescently tagged genomic constructs (S2C Fig).

IF analyses of developing retinae from the Sik fusion protein-expressing animals showed that the Siks are expressed in a specific pattern, for a limited period, during eye development. In our analyses, Sik3 expression was detected in the nucleus, only in late L3 stage eye discs, in photoreceptors R3/R4 for the first few rows after the MF, and in many other neural cells in the more posterior rows. This stepwise expression of Sik3 in PRs suggests a potential role of Sik3 in PR specification, especially in R3 and R4 (Fig 4H, 4J and 4J′). We noted some overlap between Sik3-expressing cells and Delta-expressing cells at this stage, which sets the stage for a genetic interaction.

In late L3 eye discs, Sik2::eGFP expression could be detected in center of each ommatidial cluster, starting 6–7 rows posterior to the MF, localized in the cytoplasm of photoreceptors, adjacent to the AJs in larval stage, and basal to the AJs at the pupal stage (Fig 4D–4G). This localization can be particularly meaningful. A previous report showed that AJs can be regulated by Sik3 and Lkb-1, the major regulator of Siks. It has been reported that, in mutants of Lkb1 and Sik3, AJs are defective, and Armadillo is mislocalized [84]. The detection of Sik2::eGFP not directly at the AJs but adjacent to AJs suggests the possibility that Sik2 may also have a developmental function involving regulation of cell-cell junctions, at early stages. The cytoplasmic rather than nuclear localization of Sik2 suggests that its nuclear targets such as HDACs [21] and CRTCs [9] may not be relevant in the eye at the times we examined.

Although Sik expression in earlier periods could not be assessed due to low levels of signal, we cannot exclude the possibility that they are expressed at an earlier stage like L2. So, the genetic interaction between Siks and Notch pathway members, or effectors, could be happening in L2, when D-V polarization and eye morphology are established. This could also explain how both Sik2 and Sik3 are able to modulate the Serrate and Fringe eye size phenotypes.

Our tagged Sik2 and Sik3 proteins were not expressed in the same subcellular compartments, or in the same cells at the same time. While this seems not to support the idea of redundancy between them, it is possible for tissues to adapt to the loss of one protein by upregulation of another. Compensatory mechanisms between Sik homologs and a molecular pathway involving feedback loops could explain the similarity in phenotypes observed for Sik2 and Sik3, despite their expression patterns being different during eye development (Fig 4). Also, we cannot eliminate the risk that the BAC clones used here do not contain all the enhancers necessary to recapitulate the endogenous expression patterns of the Sik proteins. Thus, the Sik expression patterns described here using tagged BAC transgenic lines would benefit from verification, if antibodies that work in immunofluorescent stainings can be developed.

Taken together, this study shows that fly Sik2 and Sik3 can promote tumor formation, that proper regulation of Sik2 and Sik3 is required for normal eye development, and that Siks functionally interact with the Notch pathway. Further studies will be necessary to uncover the molecular basis of this interaction, and to discover at which stages during development their interaction with the Notch signaling pathway takes place.

## Supporting information

S1 Fig Larval development of *Drosophila* retina. (A-C) Schematic drawings of eye-antennal disc during development. (A) During the early second instar larval stage, the posterior part of the epithelium is specified as eye precursor, via Notch signaling (shown in orange); the anterior part is specified as antennae via EGFR. (B) During late second instar stage, dorsal-ventral polarity is established. Notch receptor is expressed in the midline cells (orange). Notch is activated by Delta ligand on the dorsal half and by Serrate ligand on the ventral half. Fringe is expressed on the ventral half and suppresses the Notch activation by Serrate anywhere else than the dorsal-ventral border. (C) During the late third instar stage, the morphogenetic furrow (MF) sweeps the eye disc from posterior to anterior and initiates retinal cell specification. Delta ligand is expressed behind the MF to induce neurogenesis. Anterior is to the left, ventral is down in all drawings. The drawings are not to scale.
(EPS)

S2 Fig The Sik transgenic lines generated in this study. (A) The protein organization and genetic constructs of *Drosophila* salt inducible kinases. Sik2 and Sik3 protein kinase domains are shown in red, the UBA domains in green, and the key residues for suppression of Siks by PKA (S1032 and S563, respectively) are shown in blue. *Sik2*::*eGFP* and *Sik3*::*mCherry* are translational fusions, generated on BAC clones, where fluorescent proteins are added prior to the stop codon, after a flexible linker. Both clones comprise all the introns, exons, UTRs, and sufficient regulatory regions (14 kb and 6 kb for *Sik2* and *Sik3* respectively) to reflect the endogenous expression. UAS-Sik3::T2A::mCherry was generated using the EST clone encoding Sik3 CDS isoform A. mCherry was attached after a self-cleaving T2A linker. *Sik3*^*Δ109*^ allele was generated by excision of P element *P{EPgy2}CG42856*^*EY14354*^. Mobilization created a deletion of 9.7 kb from exon 2–10. *CG15071* gene in the intron 2 was excised, *CG42855*, which overlaps with the 5' of *Sik3*, remained intact. (B) Western blot on the wild type (*w*^*1118*^) strain crude protein extract from adult head, adult body, larval CNS (brain, ventral nerve cord, and eye-antennal imaginal discs) and larval body. >75 kD band was revealed with the α-human SIK3 antibody (estimated Sik3 size ~77 kD). Actin was used as loading control. (C) Western blot of fly head extract, from control (*w*^*1118*^) and ectopic expression of Sik3 by eye-specific drivers *(ey-GAL4*, *lGMR-GAL4 / +; UAS-Sik3*::*T2A*::*mCherry / +*), revealed with α-DsRed antibody. The majority of the mCherry was already cleaved from the fusion protein, as seen at >25 kD, and a small portion was still attached to Sik3, as seen at >100 kD (estimated size ~27 kD and 115 kD respectively).
(TIF)

S3 Fig Eye clones of Sik3 mutant. Since *Sik3* null mutants are early stage lethal, eye-specific null mutant clones were generated. (A-A′′) Full eye mutant for *Sik3* obtained by mitosis-dependent flippase, selected against *GMR-hid*. (A) Control of the eye specific pro-apoptotic element *GMR-hid* allele (Control-hid). (A′) Control of the *flippase* allele (Control-Flp). (A′′) Full eye clone of Sik3 null mutant (*Sik3*^*Δ109*^). (B-B′) Mitotic clones for *Sik3* null mutant. (B) Control of clones with *GFP* allele (Control). (B′) Mitotic clones (*Sik3*^*Δ109*^ Clones). Red region is heterozygous with one copy of GFP and one copy of Sik3 null mutant (*Sik3*^*Δ109*^ / *GMR-myrGFP*). White region is the clone, homozygous for Sik3 null mutation (*Sik3*^*Δ109*^). The full genotypes are listed in S2 Table. In all pictures, anterior is to the left, ventral is down.
(TIF)

S4 Fig Salt inducible kinase target sequences. (A) Protein sequences experimentally shown to be phosphorylated by Sik homologs were listed and aligned. 15 residues spanning the phosphorylated amino acid are shown in the figure. The *Homo sapiens* proteins start with letter ‘h’, *Mus musculus* proteins start with letter ‘m’, *Drosophila melanogaster* proteins start with letter ‘d’. Sakamototide is a synthetic peptide based on CRTC2. The index number of the residue to be phosphorylated by Sik is written after the underscore. ‘S’ stands for serine, ‘T’ stands for threonine. (B) The motif was determined by 80% likelihood consensus sequence: [L/W]X[R/K]XX[S/T]*XXXL (* marks the phosphorylated serine / threonine residue). (C) The motif was scanned against the *Drosophila melanogaster* proteome. Notch, Delta and Serrate proteins contain the consensus sequence to be phosphorylated by Sik. The serine ‘S’ or threonine ‘T’ residue to be phosphorylated is highlighted with turquoise and counted as 0. The positively charged amino acids ‘R, K’ at -3 are colored red. Leucine ‘L’ and Tryptophan ‘W’ residues at -5 and +4 are colored green.
(EPS)

S5 Fig Siks are conserved in evolution. Pairwise comparison of *Drosophila melanogaster* and *Homo sapiens* SIK2 and SIK3 proteins by global alignment. Fly Sik3-PA, the short isoform of 702 residue-long was selected for comparison. Kinase domains are highlighted with pink, the critical lysine residues in kinase domain (SIK2^K170^, SIK3^K70^) are highlighted in red, the Lkb-1 target in T-loop (SIK2^T296^, SIK3^T196^) are highlighted with yellow, the ubiquitin associated domains (UBA) were highlighted in green, the PKA target serine (SIK2^S1032A^, SIK3^S563A^) are highlighted in blue. Siks, especially the kinase domains are highly conserved in evolution. Human SIK2 and fly Sik2 kinase domains are 88.9% similar; human SIK3 and fly Sik3 domains are 85.3% similar; fly Sik2 and fly Sik3 domains are 82.5% similar at the protein level.
(PDF)

S1 Table Eye phenotypes in sensitized and eyeful background. The eye phenotype quantification (A-A′) in the “sensitized” (*Delta* overexpression) and (B-B′) in the “eyeful” backgrounds (*Delta* overexpression in combination with *GS88A8* epigenetic regulator mutation). Percentages for different backgrounds are (A,B) listed in the table and (A′,B′) shown in the histogram. The baseline eyes are similar to the sensitized parents’, which is slightly bigger than the wild type flies. The affected eyes are subclassified as fold / overgrowth (bigger eyes with at least one fold, or overgrowth of the eye), ectopic-eyes (ectopic eye tissue on the head surface or split-eyes on one side of the head), eye loss (total loss of the eye tissue), and metastasis (ectopic eye tissue in the body or in the head, which is not exposed on the surface). The incidences of normal eyes and affected eyes were quantified, and the ratio over the total number of eyes is presented in the table. (A,B) The total ratio of affected eyes is shown in the last column for each background, together with the standard error of the mean obtained from 3 independent trials. The genotypes are as in Fig 1. The full genotypes are listed in S2 Table.
(PDF)

S2 Table The full genotypes used in this study. (DOCX)

S3 Table Potential Sik targets in *Drosophila* proteome. This data is supplied in an Excel table. Notch pathway members are marked with pink. Other interesting targets are marked with orange.
(XLSX)

S1 Data (PDF)

## References

[1] Zhang Y, Gao W, Yang K, Tao H, Yang H. Salt-Inducible Kinase 1 (SIK1) is Induced by Alcohol and Suppresses Microglia Inflammation via NF-kappaB Signaling. Cell Physiol Biochem. 2018;47(4):1411–21. 10.1159/000490831 29929190

[2] Bossuyt W, De Geest N, Aerts S, Leenaerts I, Marynen P, Hassan BA. The atonal proneural transcription factor links differentiation and tumor formation in *Drosophila*. PLoS Biol. 2009;7(2):e40 10.1371/journal.pbio.1000040 19243220 PMC2652389

[3] Kumar JP, Moses K. EGF receptor and Notch signaling act upstream of Eyeless/Pax6 to control eye specification. Cell. 2001;104(5):687–97. 10.1016/s0092-8674(01)00265-3 11257223

[4] Dominguez M, de Celis JF. A dorsal/ventral boundary established by Notch controls growth and polarity in the *Drosophila* eye. Nature. 1998;396(6708):276–8. 10.1038/24402 9834035

[5] Sato A, Tomlinson A. Dorsal-ventral midline signaling in the developing *Drosophila* eye. Development. 2007;134(4):659–67. 10.1242/dev.02786 17215299

[6] Voas MG, Rebay I. Signal integration during development: insights from the *Drosophila* eye. Dev Dyn. 2004;229(1):162–75. 10.1002/dvdy.10449 14699588

[7] Tepass U, Harris KP. Adherens junctions in *Drosophila* retinal morphogenesis. Trends Cell Biol. 2007;17(1):26–35. 10.1016/j.tcb.2006.11.006 17134901

[8] Şahin HB, Çelik A. *Drosophila* Eye Development and Photoreceptor Specification. eLS 2013.

[9] Choi S, Kim W, Chung J. *Drosophila* Salt-inducible Kinase (SIK) Regulates Starvation Resistance through cAMP-response Element-binding Protein (CREB)-regulated Transcription Coactivator (CRTC). J Biol Chem. 2011;286(4):2658–64. 10.1074/jbc.C110.119222 21127058 PMC3024761

[10] Lizcano JM, Goransson O, Toth R, Deak M, Morrice NA, Boudeau J, et al LKB1 is a master kinase that activates 13 kinases of the AMPK subfamily, including MARK/PAR-1. EMBO J. 2004;23(4):833–43. 10.1038/sj.emboj.7600110 14976552 PMC381014

[11] Screaton RA, Conkright MD, Katoh Y, Best JL, Canettieri G, Jeffries S, et al The CREB coactivator TORC2 functions as a calcium- and cAMP-sensitive coincidence detector. Cell. 2004;119(1):61–74. 10.1016/j.cell.2004.09.015 15454081

[12] Horike N, Takemori H, Katoh Y, Doi J, Min L, Asano T, et al Adipose-specific expression, phosphorylation of Ser794 in insulin receptor substrate-1, and activation in diabetic animals of salt-inducible kinase-2. J Biol Chem. 2003;278(20):18440–7. 10.1074/jbc.M211770200 12624099

[13] Uebi T, Itoh Y, Hatano O, Kumagai A, Sanosaka M, Sasaki T, et al Involvement of SIK3 in Glucose and Lipid Homeostasis in Mice. PLoS One. 2012;7(5):e37803 10.1371/journal.pone.0037803 22662228 PMC3360605

[14] Sakamoto K, Bultot L, Goransson O. The Salt-Inducible Kinases: Emerging Metabolic Regulators. Trends Endocrinol Metab. 2018;29(12):827–40. 10.1016/j.tem.2018.09.007 30385008

[15] Henriksson E, Jones HA, Patel K, Peggie M, Morrice NA, Sakamoto K, et al The AMP activated protein kinase (AMPK) -related kinase Salt-inducible Kinase (SIK) 2 is Regulated by cAMP via Phosphorylation at Ser358 in Adipocytes. Biochem J. 2012. 10.1042/BJ20111932 PMC3631101 22462548

[16] Henriksson E, Sall J, Gormand A, Wasserstrom S, Morrice NA, Fritzen AM, et al SIK2 regulates CRTCs, HDAC4 and glucose uptake in adipocytes. J Cell Sci. 2015;128(3):472–86. 10.1242/jcs.153932 25472719 PMC4311129

[17] Patel K, Foretz M, Marion A, Campbell DG, Gourlay R, Boudaba N, et al The LKB1-salt-inducible kinase pathway functions as a key gluconeogenic suppressor in the liver. Nat Commun. 2014;5:4535 10.1038/ncomms5535 25088745 PMC4143937

[18] Teesalu M, Rovenko BM, Hietakangas V. Salt-Inducible Kinase 3 Provides Sugar Tolerance by Regulating NADPH/NADP+ Redox Balance. Curr Biol. 2017;27(3):458–64. 10.1016/j.cub.2016.12.032 28132818

[19] Du J, Chen Q, Takemori H, Xu H. SIK2 can be activated by deprivation of nutrition and it inhibits expression of lipogenic genes in adipocytes. Obesity (Silver Spring). 2008;16(3):531–8. 18239551 10.1038/oby.2007.98

[20] Choi S, Lim DS, Chung J. Feeding and Fasting Signals Converge on the LKB1-SIK3 Pathway to Regulate Lipid Metabolism in *Drosophila*. PLoS Genet. 2015;11(5):e1005263 10.1371/journal.pgen.1005263 25996931 PMC4440640

[21] Wang B, Moya N, Niessen S, Hoover H, Mihaylova MM, Shaw RJ, et al A hormone-dependent module regulating energy balance. Cell. 2011;145(4):596–606. 10.1016/j.cell.2011.04.013 21565616 PMC3129781

[22] Sasaki T, Takemori H, Yagita Y, Terasaki Y, Uebi T, Horike N, et al SIK2 Is a Key Regulator for Neuronal Survival after Ischemia via TORC1-CREB. Neuron. 2011;69(1):106–19. 10.1016/j.neuron.2010.12.004 21220102

[23] Kuser-Abali G, Ozcan F, Ugurlu A, Uysal A, Fuss SH, Bugra-Bilge K. SIK2 is involved in the negative modulation of insulin-dependent muller cell survival and implicated in hyperglycemia-induced cell death. Invest Ophthalmol Vis Sci. 2013;54(5):3526–37. 10.1167/iovs.12-10729 23599336

[24] Yang FC, Tan BC, Chen WH, Lin YH, Huang JY, Chang HY, et al Reversible Acetylation Regulates Salt-Inducible Kinase (SIK2) and Its Function in Autophagy. J Biol Chem. 2013. 10.1074/jbc.M112.431239 PMC3585058 23322770

[25] Chen H, Huang S, Han X, Zhang J, Shan C, Tsang YH, et al Salt-inducible kinase 3 is a novel mitotic regulator and a target for enhancing antimitotic therapeutic-mediated cell death. Cell Death Dis. 2014;5:e1177 10.1038/cddis.2014.154 24743732 PMC4001308

[26] Hayasaka N, Hirano A, Miyoshi Y, Tokuda IT, Yoshitane H, Matsuda J, et al Salt-inducible kinase 3 regulates the mammalian circadian clock by destabilizing PER2 protein. Elife. 2017;6. 10.7554/eLife.24779 PMC5747517 29227248

[27] Funato H, Miyoshi C, Fujiyama T, Kanda T, Sato M, Wang Z, et al Forward-genetics analysis of sleep in randomly mutagenized mice. Nature. 2016;539(7629):378–83. 10.1038/nature20142 27806374 PMC6076225

[28] Maduzia LL, Roberts AF, Wang H, Lin X, Chin LJ, Zimmerman CM, et al C. elegans serine-threonine kinase KIN-29 modulates TGFbeta signaling and regulates body size formation. BMC Dev Biol. 2005;5:8 10.1186/1471-213X-5-8 15840165 PMC1112587

[29] Parsons LM, Grzeschik NA, Amaratunga K, Burke P, Quinn LM, Richardson HE. A Kinome RNAi Screen in *Drosophila* Identifies Novel Genes Interacting with Lgl, aPKC, and Crb Cell Polarity Genes in Epithelial Tissues. G3 (Bethesda). 2017;7(8):2497–509. 28611255 10.1534/g3.117.043513 PMC5555457

[30] Wehr MC, Holder MV, Gailite I, Saunders RE, Maile TM, Ciirdaeva E, et al Salt-inducible kinases regulate growth through the Hippo signalling pathway in *Drosophila*. Nat Cell Biol. 2012. 10.1038/ncb2658 PMC3749438 23263283

[31] Sall J, Pettersson AM, Bjork C, Henriksson E, Wasserstrom S, Linder W, et al Salt-inducible kinase 2 and -3 are downregulated in adipose tissue from obese or insulin-resistant individuals: implications for insulin signalling and glucose uptake in human adipocytes. Diabetologia. 2017;60(2):314–23. 10.1007/s00125-016-4141-y 27807598 PMC6518086

[32] Proschel C, Hansen JN, Ali A, Tuttle E, Lacagnina M, Buscaglia G, et al Epilepsy-causing sequence variations in SIK1 disrupt synaptic activity response gene expression and affect neuronal morphology. Eur J Hum Genet. 2017;25(2):216–21. 10.1038/ejhg.2016.145 27966542 PMC5255945

[33] Ahmed AA, Lu Z, Jennings NB, Etemadmoghadam D, Capalbo L, Jacamo RO, et al SIK2 is a centrosome kinase required for bipolar mitotic spindle formation that provides a potential target for therapy in ovarian cancer. Cancer Cell. 2010;18(2):109–21. 10.1016/j.ccr.2010.06.018 20708153 PMC3954541

[34] Bon H, Wadhwa K, Schreiner A, Osborne M, Carroll T, Ramos-Montoya A, et al Salt-inducible kinase 2 regulates mitotic progression and transcription in prostate cancer. Mol Cancer Res. 2015;13(4):620–35. 10.1158/1541-7786.MCR-13-0182-T 25548099 PMC4383640

[35] Liu Y, Gao S, Chen X, Liu M, Mao C, Fang X. Overexpression of miR-203 sensitizes paclitaxel (Taxol)-resistant colorectal cancer cells through targeting the salt-inducible kinase 2 (SIK2). Tumour Biol. 2016;37(9):12231–9. 10.1007/s13277-016-5066-2 27236538

[36] Zohrap N, Saatci O, Ozes B, Coban I, Atay HM, Battaloglu E, et al SIK2 attenuates proliferation and survival of breast cancer cells with simultaneous perturbation of MAPK and PI3K/Akt pathways. Oncotarget. 2018;9(31):21876–92. 10.18632/oncotarget.25082 29774109 PMC5955149

[37] Du WQ, Zheng JN, Pei DS. The diverse oncogenic and tumor suppressor roles of salt-inducible kinase (SIK) in cancer. Expert Opin Ther Targets. 2016;20(4):477–85. 10.1517/14728222.2016.1101452 26549013

[38] Hirabayashi S, Cagan RL. Salt-inducible kinases mediate nutrient-sensing to link dietary sugar and tumorigenesis in *Drosophila*. Elife. 2015;4:e08501 10.7554/eLife.08501 26573956 PMC4643014

[39] Amara S, Majors C, Roy B, Hill S, Rose KL, Myles EL, et al Critical role of SIK3 in mediating high salt and IL-17 synergy leading to breast cancer cell proliferation. PLoS One. 2017;12(6):e0180097 10.1371/journal.pone.0180097 28658303 PMC5489190

[40] Jukam D, Desplan C. Binary regulation of Hippo pathway by Merlin/NF2, Kibra, Lgl, and Melted specifies and maintains postmitotic neuronal fate. Dev Cell. 2011;21(5):874–87. 10.1016/j.devcel.2011.10.004 22055343 PMC3215849

[41] Warming S, Costantino N, Court DL, Jenkins NA, Copeland NG. Simple and highly efficient BAC recombineering using galK selection. Nucleic Acids Res. 2005;33(4):e36 10.1093/nar/gni035 15731329 PMC549575

[42] Wang AH, Kruhlak MJ, Wu J, Bertos NR, Vezmar M, Posner BI, et al Regulation of histone deacetylase 4 by binding of 14-3-3 proteins. Mol Cell Biol. 2000;20(18):6904–12. 10.1128/mcb.20.18.6904-6912.2000 10958686 PMC88766

[43] Takemori H, Katoh Hashimoto Y, Nakae J, Olson EN, Okamoto M. Inactivation of HDAC5 by SIK1 in AICAR-treated C2C12 myoblasts. Endocr J. 2009;56(1):121–30. 10.1507/endocrj.k08e-173 18946175

[44] van der Linden AM, Nolan KM, Sengupta P. KIN-29 SIK regulates chemoreceptor gene expression via an MEF2 transcription factor and a class II HDAC. EMBO J. 2007;26(2):358–70. 10.1038/sj.emboj.7601479 17170704 PMC1783467

[45] Katoh Y, Takemori H, Lin XZ, Tamura M, Muraoka M, Satoh T, et al Silencing the constitutive active transcription factor CREB by the LKB1-SIK signaling cascade. FEBS J. 2006;273(12):2730–48. 10.1111/j.1742-4658.2006.05291.x 16817901

[46] MacKenzie KF, Clark K, Naqvi S, McGuire VA, Noehren G, Kristariyanto Y, et al PGE(2) induces macrophage IL-10 production and a regulatory-like phenotype via a protein kinase A-SIK-CRTC3 pathway. J Immunol. 2013;190(2):565–77. 10.4049/jimmunol.1202462 23241891 PMC3620524

[47] Sakamaki J, Fu A, Reeks C, Baird S, Depatie C, Al Azzabi M, et al Role of the SIK2-p35-PJA2 complex in pancreatic beta-cell functional compensation. Nat Cell Biol. 2014;16(3):234–44. 10.1038/ncb2919 24561619 PMC4107453

[48] Miranda F, Mannion D, Liu S, Zheng Y, Mangala LS, Redondo C, et al Salt-Inducible Kinase 2 Couples Ovarian Cancer Cell Metabolism with Survival at the Adipocyte-Rich Metastatic Niche. Cancer Cell. 2016;30(2):273–89. 10.1016/j.ccell.2016.06.020 27478041

[49] Bricambert J, Miranda J, Benhamed F, Girard J, Postic C, Dentin R. Salt-inducible kinase 2 links transcriptional coactivator p300 phosphorylation to the prevention of ChREBP-dependent hepatic steatosis in mice. J Clin Invest. 2010;120(12):4316–31. 10.1172/JCI41624 21084751 PMC2993582

[50] Yoshida H, Goedert M. Phosphorylation of microtubule-associated protein tau by AMPK-related kinases. J Neurochem. 2012;120(1):165–76. 10.1111/j.1471-4159.2011.07523.x 21985311

[51] Qu C, He, Lu X, Dong L, Zhu Y, Zhao Q, et al Salt-inducible Kinase (SIK1) regulates HCC progression and WNT/beta-catenin activation. J Hepatol. 2016;64(5):1076–89. 10.1016/j.jhep.2016.01.005 26778753

[52] Berggreen C, Henriksson E, Jones HA, Morrice N, Goransson O. cAMP-elevation mediated by beta-adrenergic stimulation inhibits salt-inducible kinase (SIK) 3 activity in adipocytes. Cell Signal. 2012;24(9):1863–71. 10.1016/j.cellsig.2012.05.001 22588126

[53] Brown NP, Leroy C, Sander C. MView: a web-compatible database search or multiple alignment viewer. Bioinformatics. 1998;14(4):380–1. 10.1093/bioinformatics/14.4.380 9632837

[54] Ferres-Marco D, Gutierrez-Garcia I, Vallejo DM, Bolivar J, Gutierrez-Avino FJ, Dominguez M. Epigenetic silencers and Notch collaborate to promote malignant tumours by Rb silencing. Nature. 2006;439(7075):430–6. 10.1038/nature04376 16437107

[55] Gutierrez-Avino FJ, Ferres-Marco D, Dominguez M. The position and function of the Notch-mediated eye growth organizer: the roles of JAK/STAT and four-jointed. EMBO Rep. 2009;10(9):1051–8. 10.1038/embor.2009.140 19662079 PMC2750068

[56] Moses K, Rubin GM. Glass encodes a site-specific DNA-binding protein that is regulated in response to positional signals in the developing *Drosophila* eye. Genes Dev. 1991;5(4):583–93. 10.1101/gad.5.4.583 2010085

[57] Teesalu M, Rovenko BM, Hietakangas V. Salt-Inducible Kinase 3 Provides Sugar Tolerance by Regulating NADPH/NADP(+) Redox Balance. Curr Biol. 2017;27(3):458–64. 10.1016/j.cub.2016.12.032 28132818

[58] Dominguez M, Ferres-Marco D, Gutierrez-Avino FJ, Speicher SA, Beneyto M. Growth and specification of the eye are controlled independently by Eyegone and Eyeless in *Drosophila* melanogaster. Nat Genet. 2004;36(1):31–9. 10.1038/ng1281 14702038

[59] Frankfort BJ, Nolo R, Zhang Z, Bellen H, Mardon G. senseless repression of rough is required for R8 photoreceptor differentiation in the developing *Drosophila* eye. Neuron. 2001;32(3):403–14. 10.1016/s0896-6273(01)00480-9 11709152 PMC3122332

[60] Cook T, Pichaud F, Sonneville R, Papatsenko D, Desplan C. Distinction between color photoreceptor cell fates is controlled by Prospero in *Drosophila*. Dev Cell. 2003;4(6):853–64. 10.1016/s1534-5807(03)00156-4 12791270

[61] Domingos PM, Brown S, Barrio R, Ratnakumar K, Frankfort BJ, Mardon G, et al Regulation of R7 and R8 differentiation by the spalt genes. Dev Biol. 2004;273(1):121–33. 10.1016/j.ydbio.2004.05.026 15302602

[62] Fanto M, Mayes CA, Mlodzik M. Linking cell-fate specification to planar polarity: determination of the R3/R4 photoreceptors is a prerequisite for the interpretation of the Frizzled mediated polarity signal. Mech Dev. 1998;74(1–2):51–8. 10.1016/s0925-4773(98)00063-x 9651479

[63] Izaddoost S, Nam SC, Bhat MA, Bellen HJ, Choi KW. *Drosophila* Crumbs is a positional cue in photoreceptor adherens junctions and rhabdomeres. Nature. 2002;416(6877):178–83. 10.1038/nature720 11850624

[64] Mirkovic I, Mlodzik M. Cooperative activities of *drosophila* DE-cadherin and DN-cadherin regulate the cell motility process of ommatidial rotation. Development. 2006;133(17):3283–93. 10.1242/dev.02468 16887833

[65] Wein MN, Foretz M, Fisher DE, Xavier RJ, Kronenberg HM. Salt-Inducible Kinases: Physiology, Regulation by cAMP, and Therapeutic Potential. Trends Endocrinol Metab. 2018;29(10):723–35. 10.1016/j.tem.2018.08.004 30150136 PMC6151151

[66] Bennett D, Lyulcheva E, Cobbe N. *Drosophila* as a Potential Model for Ocular Tumors. Ocul Oncol Pathol. 2015;2015 4;1(3):190–9. 10.1159/000370155 27172095 PMC4847669

[67] Nowell CS, Radtke F. Notch as a tumour suppressor. Nat Rev Cancer. 2017;17(3):145–59. 10.1038/nrc.2016.145 28154375

[68] Aster JC, Pear WS, Blacklow SC. The Varied Roles of Notch in Cancer. Annu Rev Pathol. 2017;12:245–75. 10.1146/annurev-pathol-052016-100127 27959635 PMC5933931

[69] Fan S, Tang J, Li N, Zhao Y, Ai R, Zhang K, et al Integrative analysis with expanded DNA methylation data reveals common key regulators and pathways in cancers. NPJ Genom Med. 2019;4:2 10.1038/s41525-019-0077-8 30729033 PMC6358616

[70] Charoenfuprasert S, Yang YY, Lee YC, Chao KC, Chu PY, Lai CR, et al Identification of salt-inducible kinase 3 as a novel tumor antigen associated with tumorigenesis of ovarian cancer. Oncogene. 2011. 10.1038/onc.2011.77 21399663

[71] Bolos V, Grego-Bessa J, de la Pompa JL. Notch signaling in development and cancer. Endocr Rev. 2007;28(3):339–63. 10.1210/er.2006-0046 17409286

[72] Goodwin JM, Svensson RU, Lou HJ, Winslow MM, Turk BE, Shaw RJ. An AMPK-independent signaling pathway downstream of the LKB1 tumor suppressor controls Snail1 and metastatic potential. Mol Cell. 2014;55(3):436–50. 10.1016/j.molcel.2014.06.021 25042806 PMC4151130

[73] Yao YH, Cui Y, Qiu XN, Zhang LZ, Zhang W, Li H, et al Attenuated LKB1-SIK1 signaling promotes epithelial-mesenchymal transition and radioresistance of non-small cell lung cancer cells. Chin J Cancer. 2016;35:50 10.1186/s40880-016-0113-3 27266881 PMC4897817

[74] Bailey TL, Boden M, Buske FA, Frith M, Grant CE, Clementi L, et al MEME SUITE: tools for motif discovery and searching. Nucleic Acids Res. 2009;37(Web Server issue):W202–8. 10.1093/nar/gkp335 19458158 PMC2703892

[75] Xia B, Lin M, Dong W, Chen H, Li B, Zhang X, et al Upregulation of miR-874-3p and miR-874-5p inhibits epithelial ovarian cancer malignancy via SIK2. J Biochem Mol Toxicol. 2018;32(8):e22168 10.1002/jbt.22168 30004169

[76] Cheng H, Liu P, Wang ZC, Zou L, Santiago S, Garbitt V, et al SIK1 couples LKB1 to p53-dependent anoikis and suppresses metastasis. Sci Signal. 2009;2(80):ra35 10.1126/scisignal.2000369 19622832 PMC2752275

[77] Chern JJ, Choi KW. Lobe mediates Notch signaling to control domain-specific growth in the *Drosophila* eye disc. Development. 2002;129(17):4005–13. 12163404 10.1242/dev.129.17.4005

[78] Singh A, Tare M, Puli OR, Kango-Singh M. A glimpse into dorso-ventral patterning of the *Drosophila* eye. Dev Dyn. 2012;241(1):69–84. 10.1002/dvdy.22764 22034010 PMC6538059

[79] Baonza A, Freeman M. Notch signalling and the initiation of neural development in the *Drosophila* eye. Development. 2001;128(20):3889–98. 11641214 10.1242/dev.128.20.3889

[80] Lee G, Liang C, Park G, Jang C, Jung JU, Chung J. UVRAG is required for organ rotation by regulating Notch endocytosis in *Drosophila*. Dev Biol. 2011;356(2):588–97. 10.1016/j.ydbio.2011.06.024 21729695 PMC4414087

[81] Lee HJ, Kim MY, Park HS. Phosphorylation-dependent regulation of Notch1 signaling: the fulcrum of Notch1 signaling. BMB Rep. 2015;48(8):431–7. 10.5483/bmbrep.2015.48.8.107 26058398 PMC4576950

[82] Yahara Y, Takemori H, Okada M, Kosai A, Yamashita A, Kobayashi T, et al Pterosin B prevents chondrocyte hypertrophy and osteoarthritis in mice by inhibiting Sik3. Nat Commun. 2016;7:10959 10.1038/ncomms10959 27009967 PMC4820810

[83] Katoh Y, Takemori H, Min L, Muraoka M, Doi J, Horike N, et al Salt-inducible kinase-1 represses cAMP response element-binding protein activity both in the nucleus and in the cytoplasm. Eur J Biochem. 2004;271(21):4307–19. 10.1111/j.1432-1033.2004.04372.x 15511237

[84] Amin N, Khan A, St Johnston D, Tomlinson I, Martin S, Brenman J, et al LKB1 regulates polarity remodeling and adherens junction formation in the *Drosophila* eye. Proc Natl Acad Sci U S A. 2009;106(22):8941–6. 10.1073/pnas.0812469106 19443685 PMC2690039

