# Supplementary figures and images for: Salt inducible kinases as novel Notch interactors in the developing Drosophila retina
Source: PLoS One. 2020 Jun 15;15(6):e0234744. doi: 10.1371/journal.pone.0234744 (PMC7295197; doi:10.1371/journal.pone.0234744)

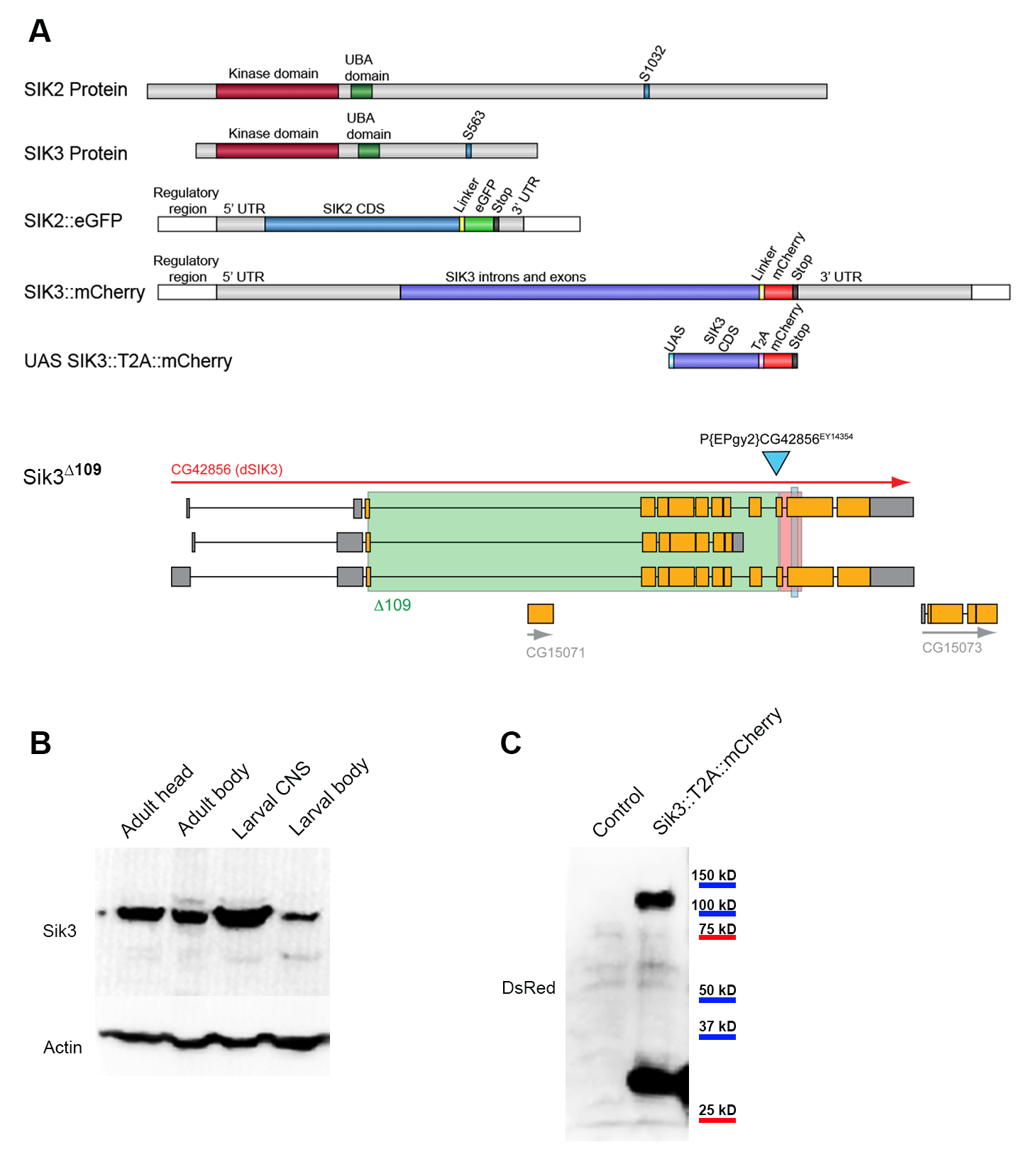

Supplement: S2 Fig — (A) The protein organization and genetic constructs of Drosophila salt inducible kinases. Sik2 and Sik3 protein kinase domains are shown in red, the UBA domains in green, and the key residues for suppression of Siks by PKA (S1032 and S563, respectively) are shown in blue. Sik2::eGFP and Sik3::mCherry are translational fusions, generated on BAC clones, where fluorescent proteins are added prior to the stop codon, after a flexible linker. Both clones comprise all the introns, exons, UTRs, and sufficient regulatory regions (14 kb and 6 kb for Sik2 and Sik3 respectively) to reflect the endogenous expression. UAS-Sik3::T2A::mCherry was generated using the EST clone encoding Sik3 CDS isoform A. mCherry was attached after a self-cleaving T2A linker. Sik3Δ109 allele was generated by excision of P element P{EPgy2}CG42856EY14354. Mobilization created a deletion of 9.7 kb from exon 2–10. CG15071 gene in the intron 2 was excised, CG42855, which overlaps with the 5' of Sik3, remained intact. (B) Western blot on the wild type (w1118) strain crude protein extract from adult head, adult body, larval CNS (brain, ventral nerve cord, and eye-antennal imaginal discs) and larval body. >75 kD band was revealed with the α-human SIK3 antibody (estimated Sik3 size ~77 kD). Actin was used as loading control. (C) Western blot of fly head extract, from control (w1118) and ectopic expression of Sik3 by eye-specific drivers (ey-GAL4, lGMR-GAL4 / +; UAS-Sik3::T2A::mCherry / +), revealed with α-DsRed antibody. The majority of the mCherry was already cleaved from the fusion protein, as seen at >25 kD, and a small portion was still attached to Sik3, as seen at >100 kD (estimated size ~27 kD and 115 kD respectively). (TIF) [file pone.0234744.s002.tif]

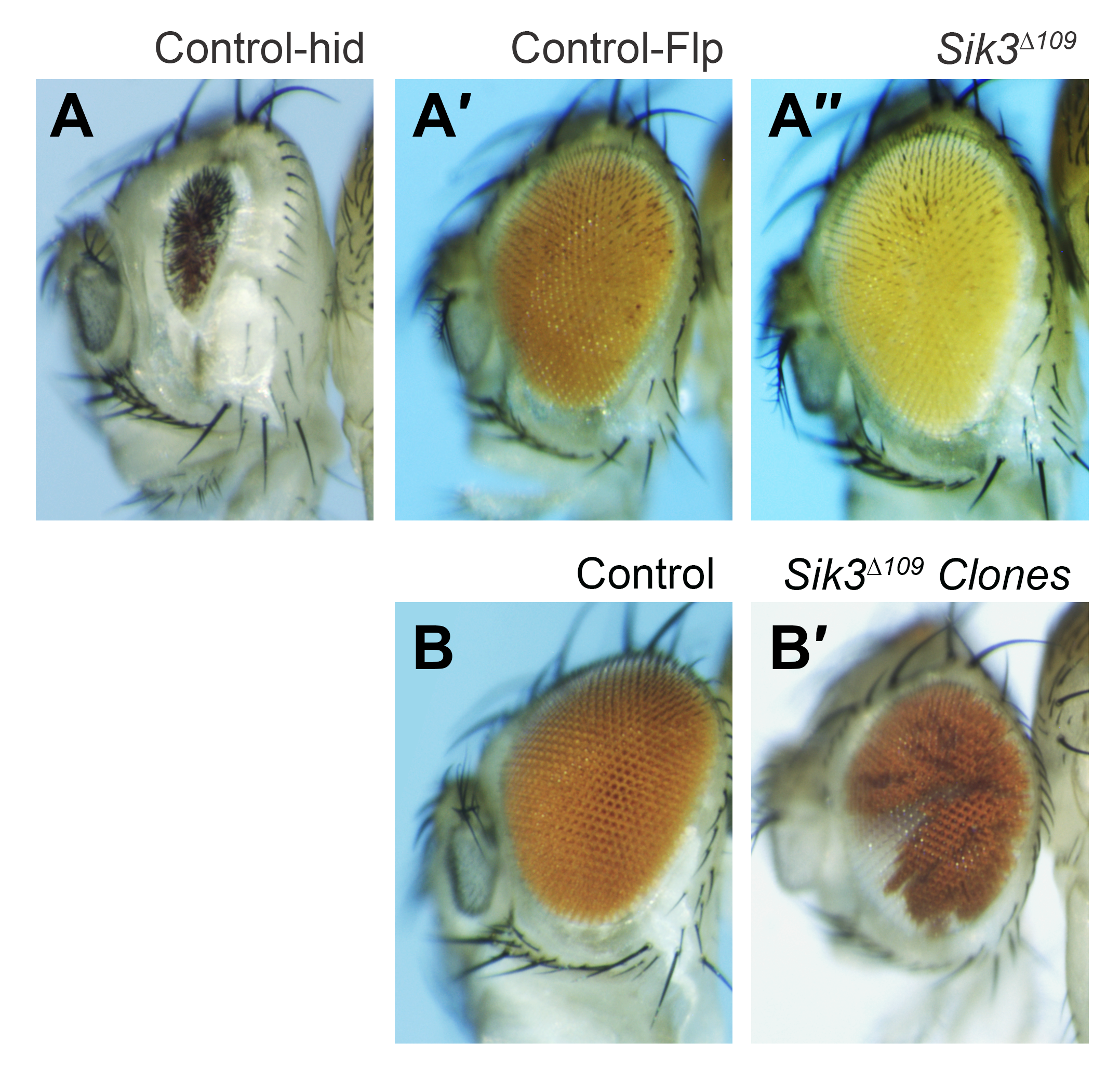

Supplement: S3 Fig — Since Sik3 null mutants are early stage lethal, eye-specific null mutant clones were generated. (A-A′′) Full eye mutant for Sik3 obtained by mitosis-dependent flippase, selected against GMR-hid. (A) Control of the eye specific pro-apoptotic element GMR-hid allele (Control-hid). (A′) Control of the flippase allele (Control-Flp). (A′′) Full eye clone of Sik3 null mutant (Sik3Δ109). (B-B′) Mitotic clones for Sik3 null mutant. (B) Control of clones with GFP allele (Control). (B′) Mitotic clones (Sik3Δ109 Clones). Red region is heterozygous with one copy of GFP and one copy of Sik3 null mutant (Sik3Δ109 / GMR-myrGFP). White region is the clone, homozygous for Sik3 null mutation (Sik3Δ109). The full genotypes are listed in S2 Table. In all pictures, anterior is to the left, ventral is down. (TIF) [file pone.0234744.s003.tif]
