# Supplementary material for: Salt inducible kinases as novel Notch interactors in the developing Drosophila retina
Source: PLoS One. 2020 Jun 15;15(6):e0234744. doi: 10.1371/journal.pone.0234744 (PMC7295197; doi:10.1371/journal.pone.0234744)
Supplement: S5 Fig — Pairwise comparison of Drosophila melanogaster and Homo sapiens SIK2 and SIK3 proteins by global alignment. Fly Sik3-PA, the short isoform of 702 residue-long was selected for comparison. Kinase domains are highlighted with pink, the critical lysine residues in kinase domain (SIK2K170, SIK3K70) are highlighted in red, the Lkb-1 target in T-loop (SIK2T296, SIK3T196) are highlighted with yellow, the ubiquitin associated domains (UBA) were highlighted in green, the PKA target serine (SIK2S1032A, SIK3S563A) are highlighted in blue. Siks, especially the kinase domains are highly conserved in evolution. Human SIK2 and fly Sik2 kinase domains are 88.9% similar; human SIK3 and fly Sik3 domains are 85.3% similar; fly Sik2 and fly Sik3 domains are 82.5% similar at the protein level. (PDF) [file pone.0234744.s005.pdf]

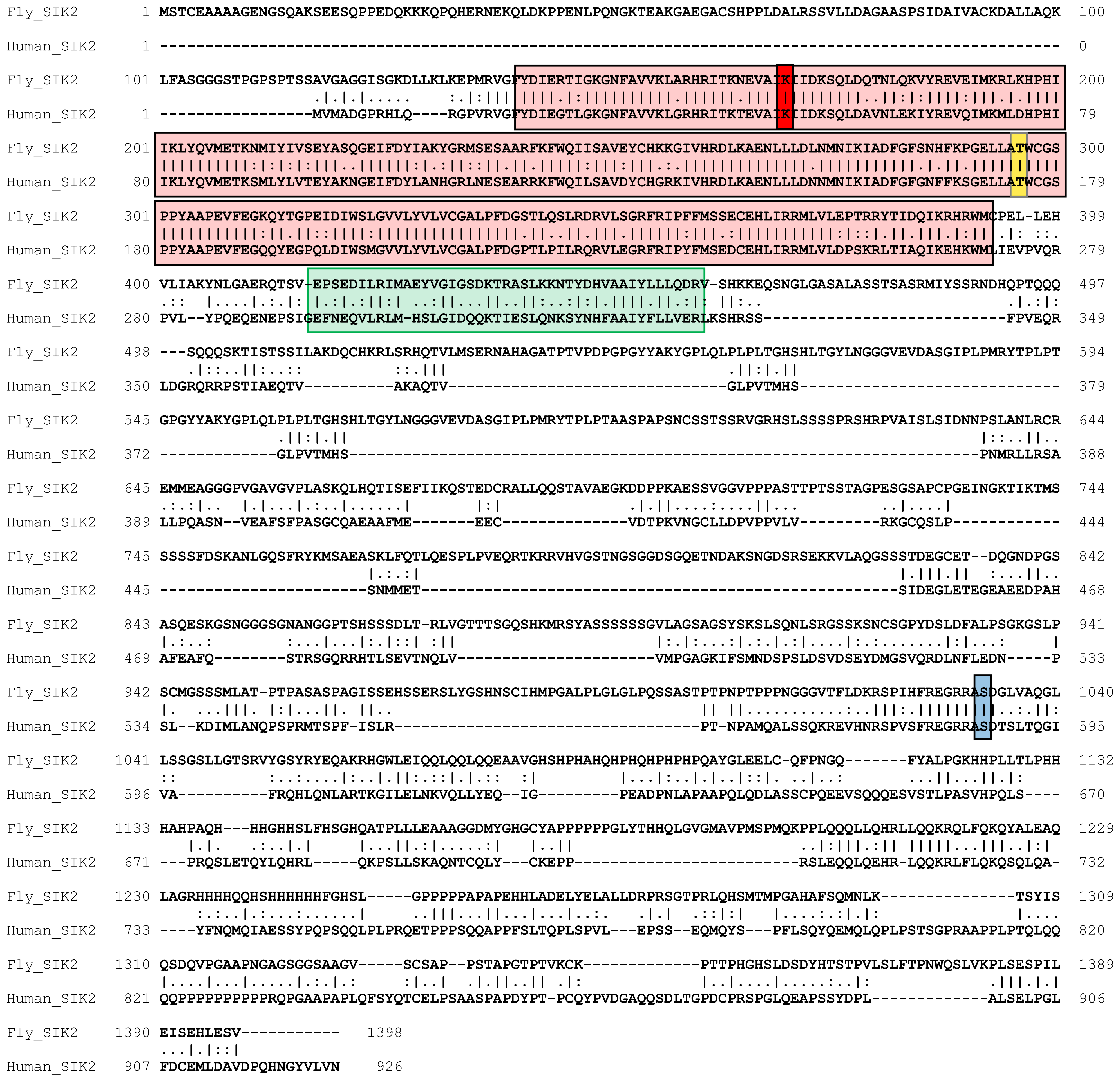
Fly_SIK2 1 MSTCEAAAAGENGSQAKSEESQPPEDQKKKQPQHERNEKQLDKPPENLPQNGKTEAKGAEGACSHPPLDALRSSVLLDAGAASPSIDAIVACKDALLAQK 100
Human_SIK2 1 -- 0
Fly_SIK2 101 LFASGGGSTPGPSPTSSAVGAGGISGKDLLKLKEPMRVGFYDIERTIGKGNFAVVKLARHRITKNEVAIKIIDKSQLDQTNLQKVYREVEIMKRLKHPHI 200
Human_SIK2 1 -----------------MVMADGPRHLQ----RGPVRVGFYDIEGTLGKGNFAVVKLGRHRITKTEVAIKIIDKSQLDAVNLEKIYREVQIMKMLDHPHI 79
Fly_SIK2 201 IKLYQVMETKNMIYIVSEYASQGEIFDYIAKYGRMSESAARFKFWQIISAVEYCHKKGIVHRDLKAENLLLDLNMNIKIADFGFSNHFKPGELLATWCGS 300
Human_SIK2 80 IKLYQVMETKSMLYLVTEYAKNGEIFDYLANHGRLNESEARRKFWQILSAVDYCHGRKIVHRDLKAENLLLDNNMNIKIADFGFGNFFKSGELLATWCGS 179
Fly_SIK2 301 PPYAAPEVFEGKQYTGPEIDIWSLGVVLYVLVCGALPFDGSTLQSLRDRVLSGRFRIPFFMSSECEHLIRRMLVLEPTRRYTIDQIKRHRWMCPEL-LEH 399
Human_SIK2 180 PPYAAPEVFEGQQYEGPQLDIWSMGVVLYVLVCGALPFDGPTLPILRQRVLEGRFRIPYFMSEDCEHLIRRMLVLDPSKRLTIAQIKEHKWMLIEVPVQR 279
Fly_SIK2 400 VLIAKYNLGAERQTSV-EPSEDILRIMAEYVGIGSDKTRASLKKNTYDHVAAIYLLLQDRV-SHKKEQSNGLGASALASSTSASRMIYSSRNDHQPTQQQ 497
Human_SIK2 280 PVL--YPQEQENEPSIGEFNEQVLRLM-HSLGIDQQKTIESLQNKSYNHFAAIYFLLVERLKSHRSS---------------------------FPVEQR 349
Fly_SIK2 498 ---SQQQSKTISTSSILAKDQCHKRLSRHQTVLMSERNAHAGATPTVPDPGPGYYAKYGPLQLPLPLTGHSHLTGYLNGGGVEVDASGIPLPMRYTPLPT 594
Human_SIK2 350 LDGRQRRPSTIAEQTV----------AKAQTV-------------------------------GLPVTMHS--------------------------- 379
Fly_SIK2 545 GPGYYAKYGPLQLPLPLTGHSHLTGYLNGGGVEVDASGIPLPMRYTPLPTAASPAPSNCSSTSSRVGRHSLSSSSPRSHRPVAISLSIDNNPSLANLRCR 644
Human_SIK2 372 -------------GLPVTMHS---PNMRLLRSA 388
Fly_SIK2 645 EMMEAGGGPVGAVGVPLASKQLHQTISEFIIKQSTEDCRALLQQSTAVAEGKDDPPKAESSVGGVPPPASTTPTSSTAGPESGSAPCPGEINGKTIKTMS 744
Human_SIK2 389 LLPQASN--VEAFSFPASGCQAEAAFME-------EEC--------------VDTPKVNGCLLDPVPPVLV---------RKGCQSLP------------ 444
Fly_SIK2 745 SSSSFDSKANLGQSFRYKMSAEASKLFQTLQESPLPVEQRTKRRVHVGSTNGSGGDSGQETNDAKSNGDSRSEKKVLAQGSSSTDEGCET--DQGNDPGS 842
Human_SIK2 445 -----------------------SNMMET---SIDEGLETEGEAEEDPAH 468
Fly_SIK2 843 ASQESKGSNGGGSGNANGGPTSHSSSDLT-RLVGTTTSGQSHKMRSYASSSSSSGVLAGSAGSYSKSLSQNLSRGSSKSNCSGPYDSLDFALPSGKGSLP 941
Human_SIK2 469 AFEAFQ--------STRSGQRRHTLSEVTNQLV---------------------VMPGAGKIFSMNDSPSLDSVDSEYDMGSVQRDLNFLEDN-----P 533
Fly_SIK2 942 SCMGSSSMLAT-PTPASASPAGISSEHSSERSLYGSHNSCIHMPGALPLGLGLPQSSASTPTPNPTPPPNGGGVTFLDKRSPIHFREGRRASDGLVAQGL 1040
Human_SIK2 534 SL--KDIMLANQPSPRMTSPF-ISLR-----------------------------------PT-NPAMQALSSQKREVHNRSPVSFREGRRASDTSLTQGI 595
Fly_SIK2 1041 LSSGSLLGTSRVYGSYRYEQAKRHGWLEIQQLQQLQQEAAVGHSHPHAHQHPHQHPHPHPQAYGLEELC-QFPNGQ-------FYALPGKHHPLLTLPHH 1132
Human_SIK2 596 VA----------FRQHLQNLARTKGILELNKVQLLYEQ--IG---------PEADPNLAPAAPQLQDLASSCPQEEVSQQQESVSTLPASVHPQLS---- 670
Fly_SIK2 1133 HAHPAQH---HHGHHSLFHSGHQATPLLLEAAAGGDMYGHGCYAPPPPPPGLYTHHQLGVGMAVPMSPMQKPPLQQQLLQHRLLQQKRQLFQKQYALEAQ 1229
Human_SIK2 671 ---PRQSLETQYLQHRL-----QKPSLLSKAQNTCQLY---CKEPP-------------------------RSLEQQLQEHR-LQQKRLFLQKQSQLQA- 732
Fly_SIK2 1230 LAGRHHHHQQHSHHHHHHFGHSL-----GPPPPPAPAPEHHLADELYELALLDRPRSGTPRLQHSMTMPGAHAFSQMNLK---------------TSYIS 1309
Human_SIK2 733 ----YFNQMQIAESSYPQPSQQLPLPRQETPPPSQQAPPFSLTQPLSPVL---EPSS--EQMQYS---PFLSQYQEMQLQPLPSTSGPRAAPPLPTQLQQ 820
Fly_SIK2 1310 QSDQVPGAAPNGAGSGGSAAGV-----SCSAP--PSTAPGTPTVKCK-------------PTTPHGHSLDSDYHTSTPVLSLFTPNWQSLVKPLSESPIL 1389
Human_SIK2 821 QQPPPPPPPPPPRQPGAAPAPLQFSYQTCELPSAASPAPDYPT-PCQYPVDGAQQSDLTGPDCPRSPGLQEAPSSYDPL-------------ALSELPGL 906
Fly_SIK2 1390 EISEHLESV----------- 1398
Human_SIK2 907 FDCEMLDAVDPQHNGYVLVN 926

```
Fly_SIK3       1 MATTPTAGPAAAPPTSSTPQNYKVPSTSKISVDKLLRVGYYELEKTIGKGNFAVVKLATNIVTKTKVAIKIIDKTCLNEEYLNKTFREIAILKSLRHPHI 100
                                                   ...|:| ||:::|||||||||||.||::|||.||| |||||.|:||.|.|.|||:.|:|.|.||||
Human_SIK3     1 ---------------------------------MPARIGYYEIDRTIGKGNFAVVKRATHLVTKAKVAIKIIDKTQLDEENLKKIFREVQIMKMLCHPHI 67

Fly_SIK3     101 TRLYEVMESQSMIYLVTEYAPNGEIFDHLVANGRMKEPEAARVFTQLVSAVHYCHRRGVVHRDLKAENVLLDKDMNIKLADFGFSNHYEEGATLKTWCGS 200
                 .|||:|||::.|||||||||..|||||||||:|||.|.||.|.|.|.|:|:||::||.|.:||||||||||:|||.::|||:|||||||.:..|..||||||
Human_SIK3    68 IRLYQVMETERMIYLVTEYASGGEIFDHLVAHGRMAEKEARRKFKQIVTAVYFCHCRNIVHRDLKAENLLLDANLNIKIADFGFSNLFTPGQLLKTWCGS 167

Fly_SIK3     201 PPYAAPEVFQGLEYDGPKSDIWSLGVVLYALVCGALPFDGKTILELKSRVVLGKFRIPFFMSQECEQLIRNMLVVEPDRRYTIKQIIKHRWLSEWQSEMQ 300
                 |||||||:|:|.||||||.|||||||||||.|||||||||.|:..|::||:.||||||||||.|||.|||:|||::|::|.:::||.||:|:
Human_SIK3   168 PPYAAPELFEGKEYDGPKVDIWSLGVVLYVLVCGALPFDGSTLQNLRARVLSGKFRIPFFMSTECEHLIRHMLVLDPNKRLSMEQICKHKWM-------- 259

Fly_SIK3     301 EQERFGDMSPGSGTVSKSASTSSLGSASDSPPQLDSVVMTHMLQLPGLTADMIAQSVHEQRFDNIYAIYNLLHDKLQQRRRENQRLQHHASLAYSRSRKT 400
                    :.||..|....:  .|.....|.......|..:.|::.  ::..||..:...||:....:|:..|||:||.|:  .:|.:..||
Human_SIK3   260 ---KLGDADPNFDRL--IAECQQLKEERQVDPLNEDVLLA--MEDMGLDKEQTLQSLRSDAYDHYSAIYSLLCDR--HKRHKTLRL-------------- 336

Fly_SIK3     401 SITTGVVDRSEPVKQESLDRLSPLSNANASSSALGFGWSDVAVDL--------EKYGDFELEC-LARSNDPPVNAQHLSAHAGGGVNGANTRRHTVGPGD 491
                      |.:    |....:|...:|: |..|..:......|...|.|        |..|...|:. ......|....::||           .||||||..|
Human_SIK3   337 ----GAL----PSMPRALAFQAPV-NIQAEQAGTAMNISVPQVQLINPENQIVEPDGTLNLDSDEGEEPSPEALVRYLS-----------MRRHTVGVAD 416

Fly_SIK3     492 VAHE-----QALANPNVPPIDFKCPPQCSDPTQPVPYYPVNLPMLQNQPLHNLTIKDQHLLKPPVVM---GASSFGRRASDGGANLHIYYPATGTVVGPA 583
                 ...|     |.|. |..|.:::.:.|.....|.  |.:....|||...||...|..|:|.||:||.:.   |....||||||||||:.::
Human_SIK3   417 PRTEVMEDLQKLL-PGFPGVNPQAPFLQVAPN--VNFMHNLLPMQNLQPTGQLEYKEQSLLQPPTLQLLNGMGPLGRRASDGGANIQLH----------- 502

Fly_SIK3     584 QGQQMDTAGYYINPNCGTDPL-----AVQELSPLNEQSVAQMQCCQENATGECNEE-LQSYMQKRGGTQRHTVGCTEDLSVAHGSGAGSQS--------Q 669
                  .||:      :....|..||     ||..::|::|          |::.||.::| :||...|...|....   ||..|.......|:.|        :
Human_SIK3   503 -AQQL------LKRPRGPSPLVTMTPAVPAVTPVDE----------ESSDGEPDQEAVQSSTYKDSNTLHLP---TERFSPVRRFSDGAASIQAFKAHLE 582

Fly_SIK3     670 APTTSSNMRQRRTGLLTVTERPPGALERLTKIL------------------------------------------------------------------- 702
                 ....:|:::|.:.           ..|:|.|:.
Human_SIK3   583 KMGNNSSIKQLQQ-----------ECEQLQKMYGGQIDERTLEKTQQQHMLYQQEQHHQILQQQIQDSICPPQPSPPLQAACENQPALLTHQLQRLRIQP 671

Fly_SIK3     703 ---------------------------------------------------------------------------------------------------- 702

Human_SIK3   672 SSPPPNHPNNHLFRQPSNSPPPMSSAMIQPHGAASSSQFQGLPSRSAIFQQQPENCSSPPNVALTCLGMQQPAQSQQVTIQVQEPVDMLSNMPGTAAGSS 771

Fly_SIK3     703 ---------------------------------------------------------------------------------------------------- 702

Human_SIK3   772 GRGISISPSAGQMQMQHRTNLMATLSYGHRPLSKQLSADSAEAHSLNVNRFSPANYDQAHLHPHLFSDQSRGSPSSYSPSTGVGFSPTQALKVPPLDQFP 871

Fly_SIK3     703 ---------------------------------------------------------------------------------------------------- 702

Human_SIK3   872 TFPPSAHQQPPHYTTSALQQALLSPTPPDYTRHQQVPHILQGLLSPRHSLTGHSDIRLPPTEFAQLIKRQQQQRQQQQQQQQQQEYQELFRHMNQGDAGS 971

Fly_SIK3     703 ---------------------------------------------------------------------------------------------------- 702

Human_SIK3   972 LAPSLGGQSMTERQALSYQNADSYHHHTSPQHLLQIRAQECVSQASSPTPPHGYAHQPALMHSESMEEDCSCEGAKDGFQDSKSSSTLTKGCHDSPLLLS 1071

Fly_SIK3     703 ---------------------------------------------------------------------------------------------------- 702

Human_SIK3  1072 TGGPGDPESLLGTVSHAQELGIHPYGHQPTAAFSKNKVPSREPVIGNCMDRSSPGQAVELPDHNGLGYPARPSVHEHHRPRALQRHHTIQNSDDAYVQLD 1171

Fly_SIK3     703 --------------------------------------------------------------------------------------------    702

Human_SIK3  1172 NLPGMSLVAGKALSSARMSDAVLSQSSLMGSQQFQDGENEECGASLGGHEHPDLSDGSQHLNSSCYPSTCITDILLSYKHPEVSFSMEQAGV    1263
```
