# Supplementary material for: Salt inducible kinases as novel Notch interactors in the developing Drosophila retina
Source: PLoS One. 2020 Jun 15;15(6):e0234744. doi: 10.1371/journal.pone.0234744 (PMC7295197; doi:10.1371/journal.pone.0234744)

A

Genotype	Sensitized background (*ey-Gal4 > Dl*)					
	Baseline	Fold / Overgrowth	Ectopic eyes	Eye loss	Metastasis	Total affected
-	98.6	1.1	0.3	0.0	0.0	**1.4 ±0.3**
>*Sik2 OE*	76.7	19.1	4.2	0.0	0.0	**23.3 ±3.5**
>$Sik2^{RNAi}$	87.9	4.3	5.1	2.5	0.2	**12.1 ±3.2**
>$Sik2^{S1032A}$	92.3	7.1	0.6	0.0	0.0	**7.7 ±0.5**
>$Sik2^{K170M}$	78.1	19.4	2.0	0.6	0.0	**21.9 ±3.0**
>*Sik3 OE*	97.9	1.5	0.4	0.0	0.2	**2.1 ±0.9**
>$Sik3^{RNAi}$	92.4	7.6	0.0	0.0	0.0	**7.6 ±1.0**
>$Sik3^{S563A}$	60.5	9.0	12.6	17.0	0.9	**39.5 ±6.1**

B

Genotype	Eyeful background (*ey-Gal4 > Dl, GS88A8*)					
	Baseline	Fold / Overgrowth	Ectopic eyes	Eye loss	Metastasis	Total affected
-	71.4	19.8	5.7	1.8	1.4	**28.6 ±11.0**
>*Sik2 OE*	67.8	13.9	10.0	6.2	2.1	**32.1 ±10.3**
>$Sik2^{RNAi}$	52.7	29.5	8.4	8.1	1.3	**47.3 ±10.1**
>$Sik2^{S1032A}$	76.4	8.8	7.2	4.4	3.2	**23.6 ±4.6**
>$Sik2^{K170M}$	67.1	17.6	45.0	6.6	3.9	**33.0 ±10.1**
>*Sik3 OE*	74.5	15.0	4.8	4.9	1.0	**25.5 ±9.2**
>$Sik3^{RNAi}$	61.5	23.9	11.9	2.6	0.2	**38.5 ±11.9**
>$Sik3^{S563A}$	55.0	21.0	9.0	14.0	1.0	**45.0 ±6.1**

A′

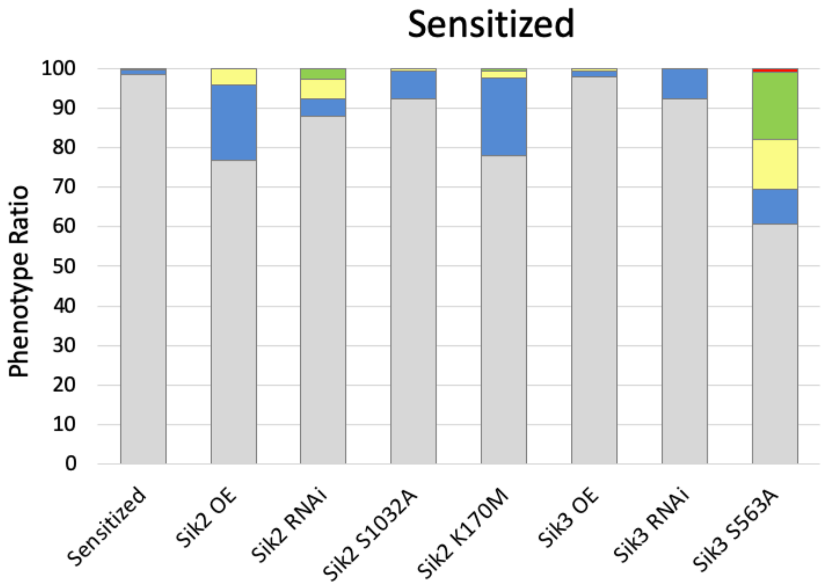

B′

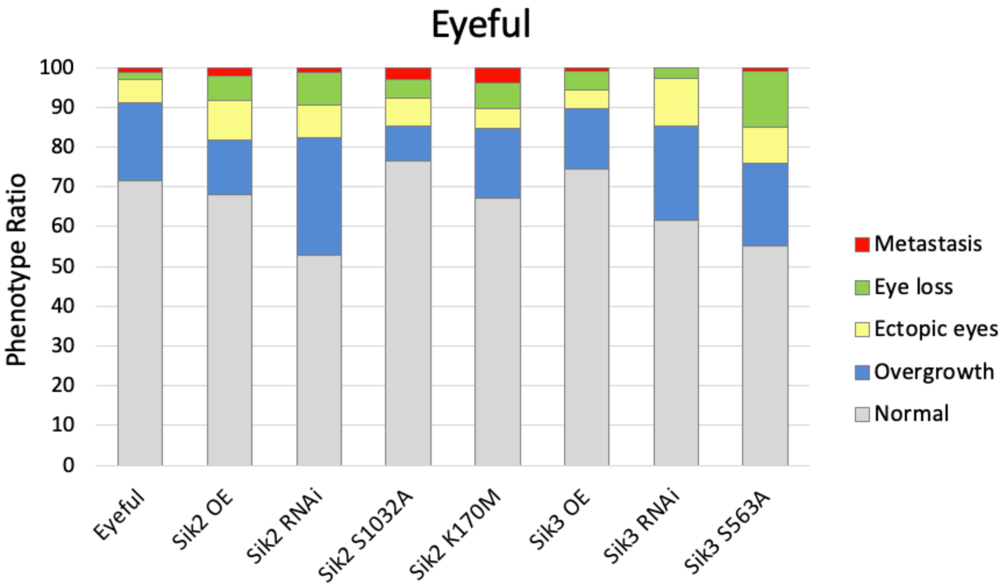

Supplement: S1 Table — The eye phenotype quantification (A-A′) in the “sensitized” (Delta overexpression) and (B-B′) in the “eyeful” backgrounds (Delta overexpression in combination with GS88A8 epigenetic regulator mutation). Percentages for different backgrounds are (A,B) listed in the table and (A′,B′) shown in the histogram. The baseline eyes are similar to the sensitized parents’, which is slightly bigger than the wild type flies. The affected eyes are subclassified as fold / overgrowth (bigger eyes with at least one fold, or overgrowth of the eye), ectopic-eyes (ectopic eye tissue on the head surface or split-eyes on one side of the head), eye loss (total loss of the eye tissue), and metastasis (ectopic eye tissue in the body or in the head, which is not exposed on the surface). The incidences of normal eyes and affected eyes were quantified, and the ratio over the total number of eyes is presented in the table. (A,B) The total ratio of affected eyes is shown in the last column for each background, together with the standard error of the mean obtained from 3 independent trials. The genotypes are as in Fig 1. The full genotypes are listed in S2 Table. (PDF) [file pone.0234744.s006.pdf]
