# Supplementary material for: Salt inducible kinases as novel Notch interactors in the developing Drosophila retina
Source: PLoS One. 2020 Jun 15;15(6):e0234744. doi: 10.1371/journal.pone.0234744 (PMC7295197; doi:10.1371/journal.pone.0234744)

This document shows data not included in the manuscript "**Salt inducible kinases as novel Notch interactors in the developing *Drosophila* retina**" by H. Bahar Şahin, Sercan Sayın, Maxine Holder, Kuyaş Buğra, Arzu Çelik to Plos One Journal in April 2020.

Above each figure, you can see the entire genotype and the description.

i) Examples of antenna enlargement and possibly duplication, caused by the Sik3 constitutively active ($Sik3^{S563A}$) in the sensitized background. Please compare with **Fig1E**.
Genotype: *+ / + ; ey-GAL4 , UAS Delta / UAS-*$Sik3^{S563A}$

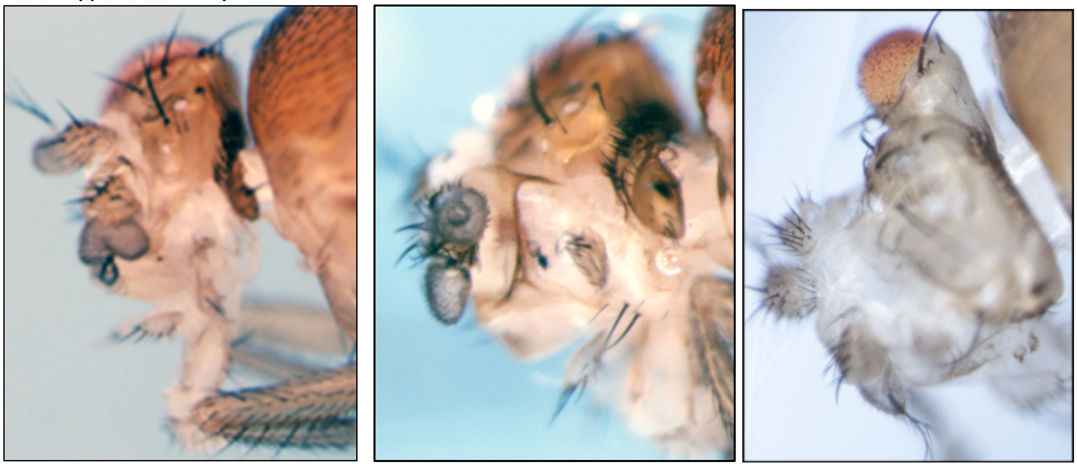

ii) Sik2 constitutively active ($Sik2^{S1032A}$) in Delta overexpressing background. An example of immense necrosis and an example of total eye loss. Please compare with **Fig3B**.
Genotype: *UAS-Dicer2 / UAS-Delta ; ey-GAL4, lGMR-GAL4 / + ; UAS-*$Sik2^{S1032A}$ */ +*

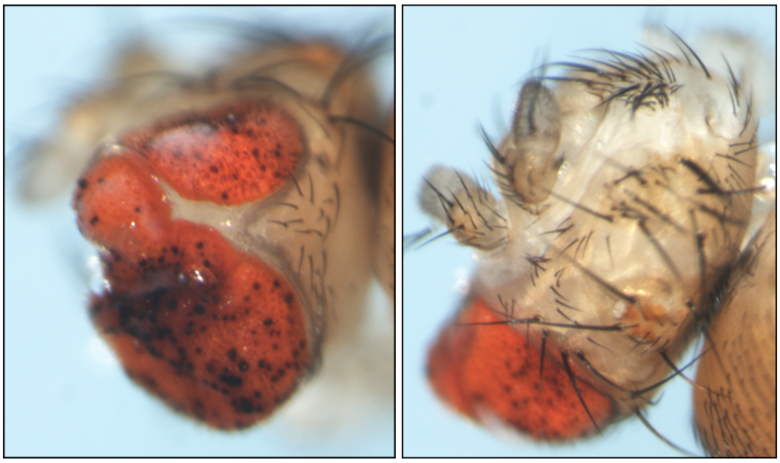

iii) Sik3 constitutively active ($Sik3^{S563A}$) in Delta overexpressing background. An example with necrotic lens and irregular retinal surface. Please compare with **Fig3E**.
Genotype: *UAS-Dicer2 / UAS-Delta ; ey-GAL4, lGMR-GAL4 / UAS-*$Sik3^{S563A}$ *; + / +*

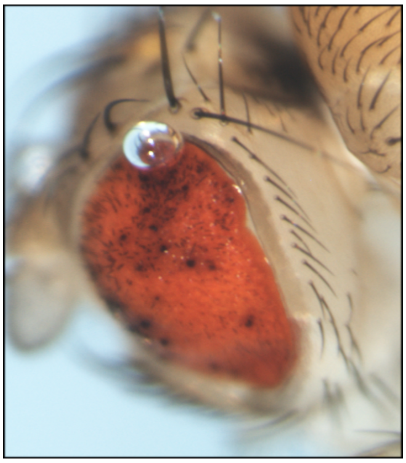

iv) Sik3 knock down (Sik3^{RNAi}) in Serrate knock down background. It doesn't alter the phenotype of control, Fig3G. Please compare with **Fig3G-L**.

Genotype: *UAS-Dicer2 / + ; ey-GAL4, lGMR-GAL4 / + ; UAS-Ser^{RNAi} / UAS-Sik3^{RNAi}*

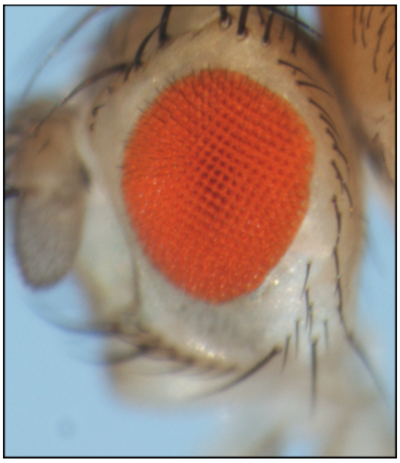

v) Sik3 knock down (Sik3^{RNAi}) in Serrate knock down background. This cross is done with another Serrate^{RNAi} allele (GD27174). With this alternative line, Sik3 knock down does not alter the control phenotype either. Please compare with **Fig3G-L**.

Genotype: *UAS-Dicer2 / + ; ey-GAL4, lGMR-GAL4 / + ; UAS-Ser$^{RNAi\ GD27174}$ / UAS-Sik3^{RNAi}*

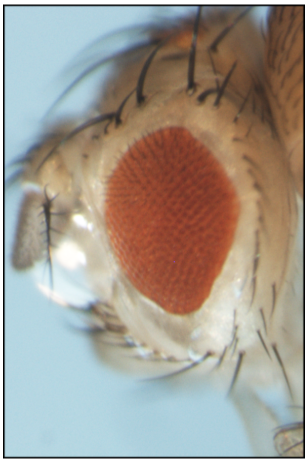

vi) Wild type Sik2 expression (Sik2 OE) in Serrate knock down background. This cross is done with an alternative Serrate^{RNAi} allele (GD27174) and ends up in the same result, a total eye loss. Please compare with **Fig3H**.

Genotype: *UAS-Dicer2 / + ; ey-GAL4, lGMR-GAL4 / + ; UAS-Ser$^{RNAi\ GD27174}$ / UAS-Sik2*

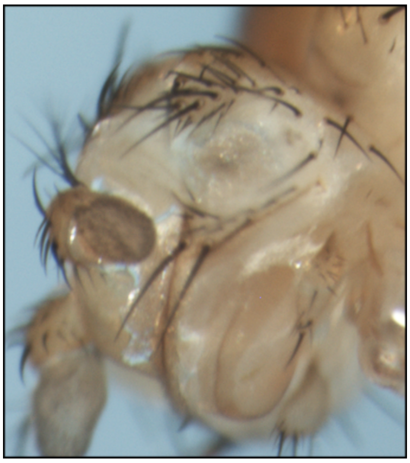

vii) Sik2 constitutively active (Sik2^{S1032A}) in Serrate knock down background. This cross is done with an alternative Serrate^{RNAi} allele (GD27174) and ends up in the same result, total eye loss. Please compare with **Fig3J**.

Genotype: *UAS-Dicer2 / + ; ey-GAL4, lGMR-GAL4 / + ; UAS-Ser$^{RNAi\ GD27174}$ / UAS-Sik2^{S1032A}*

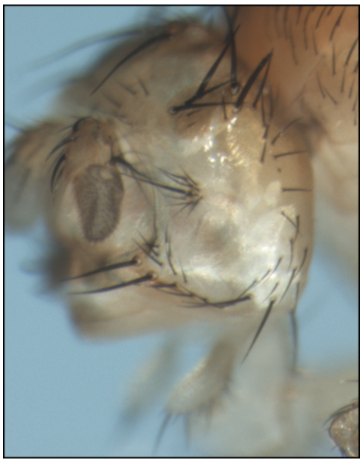

viii) Sik3 constitutively active (Sik3^{S563A}) in Serrate knock down background. This cross is done with an alternative Serrate^{RNAi} allele (GD27174) and ends up in the same result, mostly total eye loss. Please compare with **Fig3L**.

Genotype: *UAS-Dicer2 / + ; ey-GAL4, lGMR-GAL4 / UAS-Sik3^{S563A} ; UAS-Ser$^{RNAi\ GD27174}$ / +*

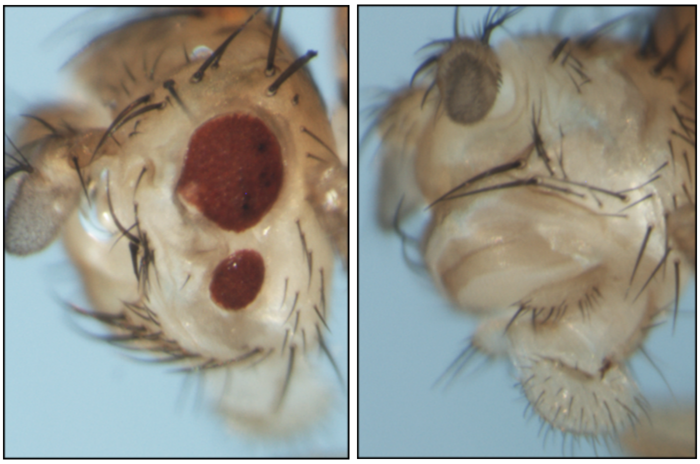

ix) Wild type Sik3 expression (Sik3 OE) in Fringe knock down background. Sik3 overexpression doesn't alter the phenotype of control, Fig3M. Please compare with **Fig3M-O**.

Genotype: *UAS-Dicer2 / + ; ey-GAL4, lGMR-GAL4 , UAS-Fng / + ; UAS-Sik3::T2A::mCherry / +*

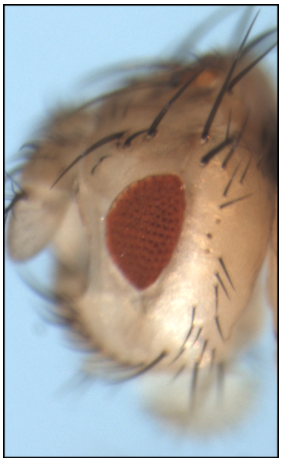

x) Delta knock down. Opposite of Delta overexpression, it causes smaller eyes. Please compare with **Fig3A**.
Genotype: *UAS-Dicer2 / + ; ey-GAL4, lGMR-GAL4 , UAS-Delta*$^{RNAi\ GD3720}$*/ + ; + / +*

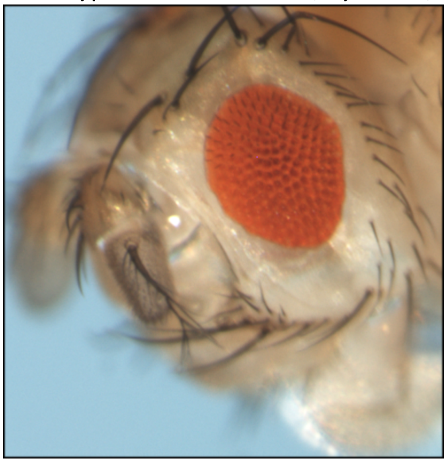

xi) Sik2 constitutively active allele (Sik2^{K170M}) in Delta knock down background. It enhances the small eyes created by the decrease in Delta protein. Please compare with the previous figure (control). This is another piece of data suggesting a genetic interaction between Sik2 and Notch signaling pathway.
Genotype: *UAS-Dicer2 / + ; ey-GAL4, lGMR-GAL4 , UAS-Delta*$^{RNAi\ GD3720}$ */ SIK2*^{K170M} *; + / +*

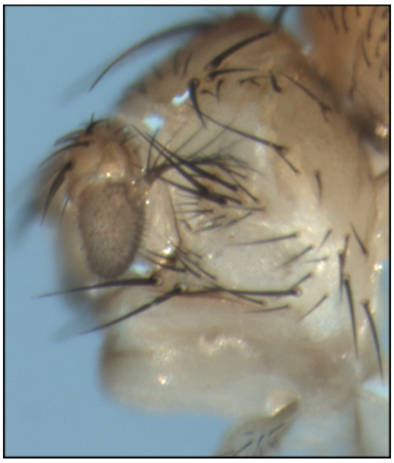

xii) The Western Blot, uncropped and unmodified version. From left to right, protein extraction from adult head, adult carcass, larval brain, VNC and eye discs, larval carcass from wild type flies. The protein ladder is loaded to the 5th lane. The two lanes on the right are irrelevant. The blot on the top is treated with rabbit α-SIK3 human antibody (ab88495) and the bottom half is treated with mouse α-Actin human antibody (CS 8H10D10). Please compare with **S2B Fig**.

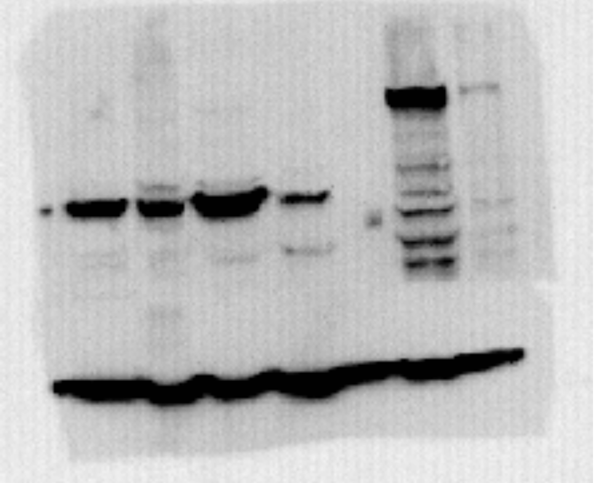

xiii) The Western Blot, uncropped and unmodified version. From left to right, the first two lanes are unrelated, the 3rd lane is the protein ladder, then comes the Sik3::T2A::mCherry overexpressing larvae and the control larvae of the driver background. It is treated with rabbit α-DsRed antibody (Clontech 632496). Please compare with **S2C Fig**.

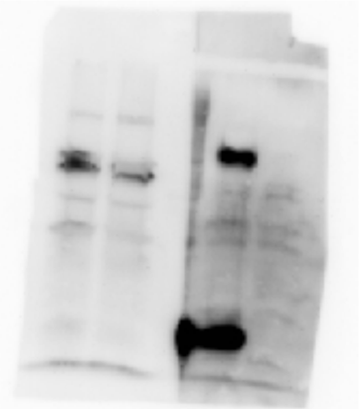

xiv) The Western Blot trying to detect SIK2.
The image on the left: From left to right, larval crude extract, adult body, adult head from the wild type flies; protein ladder loaded in the 4th lane. The three lanes on the right are unrelated (treated with α-SIK3 antibody). The left part is blotted with α-SIK2 human antibody from SantaCruz sc33074. It doesn't reveal any specific band.
The image on the right: It was tried once again with wild type fly head crude extract and the same antibody, and did not reveal any specific signal again. Please compare with **S2 Fig**.

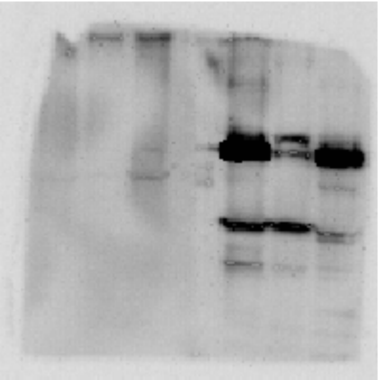

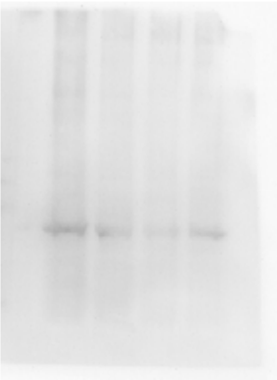

xv) The Western Blot trying to detect SIK2. The membrane is blotted against SIK2 (sc33074). It doesn't reveal 150 kD Sik2 protein, but rather reveals the short isoform of Sik3 (Sik3-PA) between 77 kD, as also seen upon overexpression (between 75-100 kD, check the green arrows).

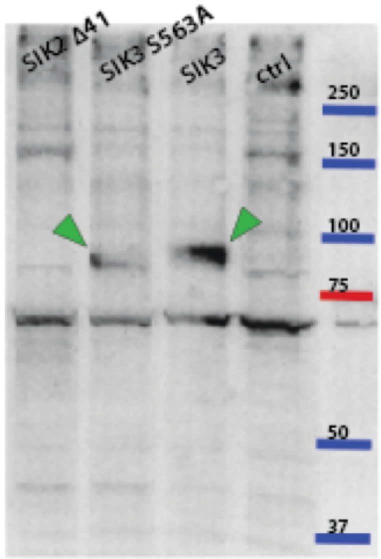

sc33074

xvi) The confocal microscopy image of wild type adult head section, stained with α-human Sik2 antibody from Life Span LS-B2068. It doesn't reveal any specific signal. Please compare with **Fig 4**.

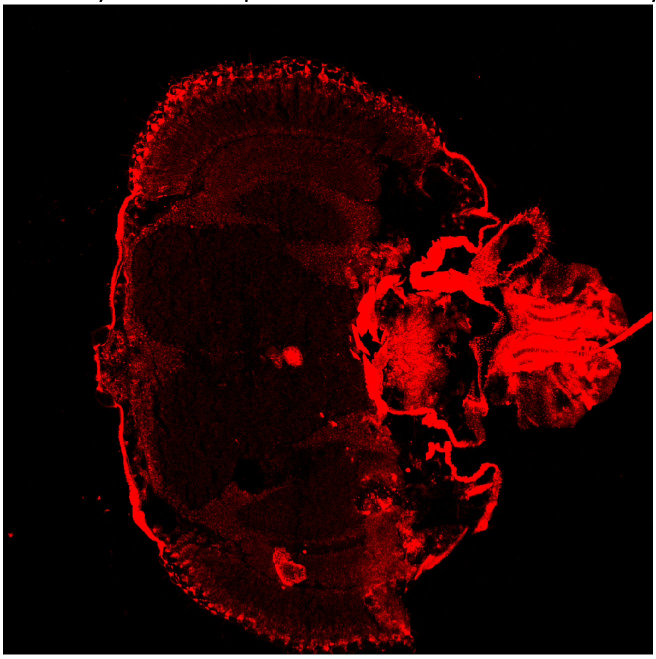

xvii) The confocal microscopy image of wild type adult head section, mostly focused on retina and optic lobe, stained with α-human Sik2 antibody from SantaCruz sc33074. It doesn't reveal any specific signal. Please compare with **Fig 4**.

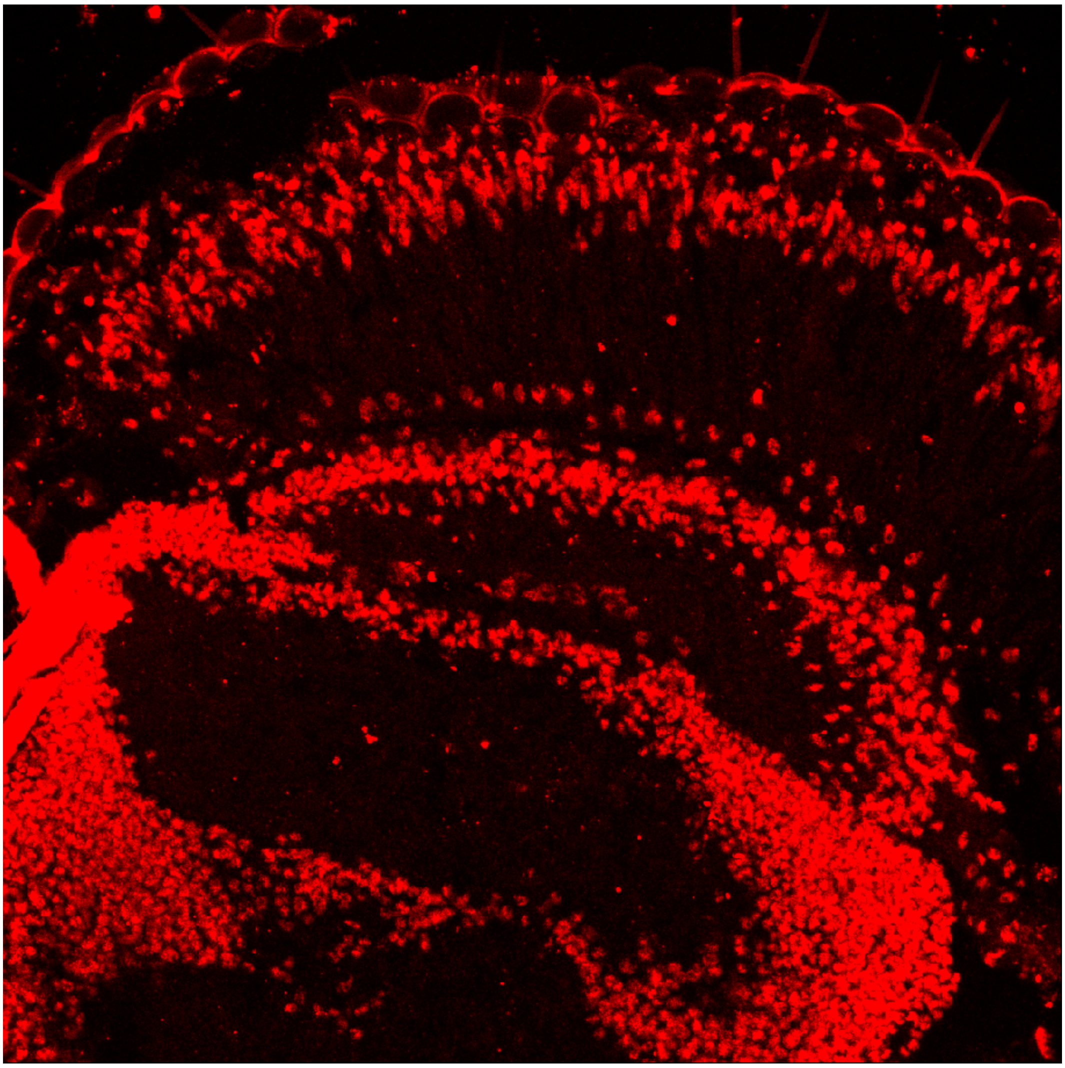

xviii) The confocal microscopy image of wild type adult head section, stained with rabbit α-SIK3 human antibody from Abcam ab88495 in red. Green shows Elav (pan-neural) and blue shows repo (pan-glial). Sik3 signal doesn't reveal any specific pattern. Please compare with **Fig 4**.

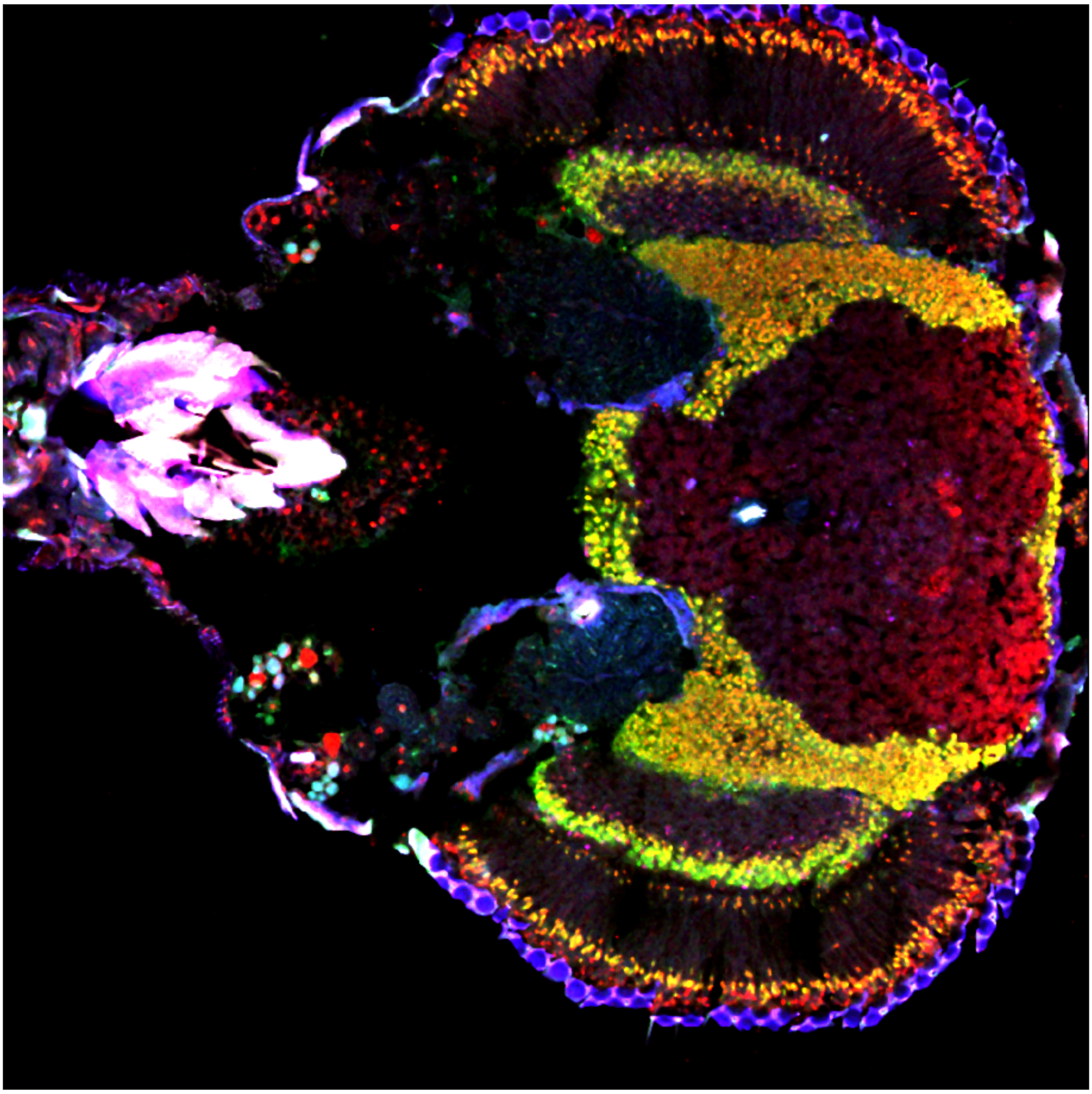

Supplement: S1 Data — (PDF) [file pone.0234744.s009.pdf]
